# New Tetramic Acid Derivatives From the Deep-Sea-Derived Fungus *Penicillium* sp. SCSIO06868 With SARS-CoV-2 M^pro^ Inhibitory Activity Evaluation

**DOI:** 10.3389/fmicb.2021.730807

**Published:** 2021-09-27

**Authors:** Xiaoyan Pang, Weihao Chen, Xin Wang, Xuefeng Zhou, Bin Yang, Xinpeng Tian, Junfeng Wang, Shihai Xu, Yonghong Liu

**Affiliations:** ^1^CAS Key Laboratory of Tropical Marine Bio-Resources and Ecology, Guangdong Key Laboratory of Marine Materia Medica, South China Sea Institute of Oceanology, Chinese Academy of Sciences (CAS), Guangzhou, China; ^2^Sanya Institute of Oceanology, SCSIO, Yazhou Scientific Bay, Sanya, China; ^3^College of Chemistry and Materials Science, Jinan University, Guangzhou, China; ^4^Center for Innovative Marine Drug Screening and Evaluation, School of Medicine and Pharmacy, Ocean University of China, Qingdao, China

**Keywords:** deep-sea-derived fungus, *Penicillium* sp., secondary metabolites, antibacterial, antiviral

## Abstract

Three new tetramic acid derivatives (**1**–**3**) and a new polyketide (**4**) along with eight known compounds (**5**–**12**) were isolated from cultures of the deep-sea-derived fungus *Penicillium* sp. SCSIO06868. Four new structures were elucidated by analysis of one-dimensional/two-dimensional nuclear magnetic resonance (NMR) data and high-resolution electrospray ionization mass spectrometry. Their absolute configurations were established by X-ray crystallography analysis and comparison of the experimental and reported electronic circular dichroism (ECD) values or specific optical rotation. Compound **3** exhibited potent, selective inhibitory activities against *Staphylococcus aureus* and methicillin-resistant *S. aureus* with minimum inhibitory concentration values of both 2.5 μg/ml. Also, compound **3** showed weak antiviral activity against severe acute respiratory syndrome coronavirus 2 main protease, which was responsible for the coronavirus disease 2019 pandemic.

## Introduction

**FIGURE 1:**
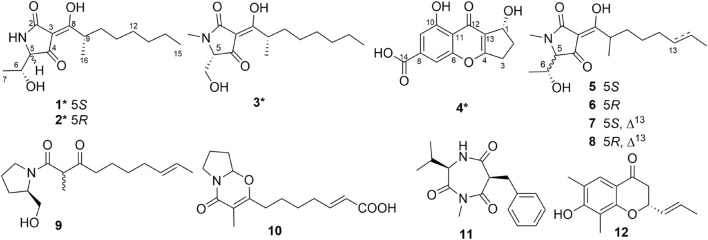
Chemical structures of compounds **1**–**12**. *Means new compounds.

Natural products bearing a tetramic acid structural fragment (pyrrolidine-2,4-dione) are isolated from various terrestrial and marine organisms, such as bacteria, cyanobacteria, fungi, and sponges (Mo et al., 2014; Jiang et al., 2020). Tetramic acids showed a remarkable diversity of bioactivities, including antitumor (Lin et al., 2008; Fan et al., 2020), antiviral (Sun et al., 2015), antibacterial (Nord et al., 2020; Wingen et al., 2020), larvicidal (Mao et al., 2019), and herbicidal (Schrey et al., 2019) activities (Schobert and Schlenk, 2008; Mo et al., 2014; Jiang et al., 2020). Among the different marine sources, marine fungi mainly containing *Aspergillus*, *Penicillium*, and *Cladosporium* species are the dominant sources of the rapidly increasing numbers of tetramic acids (Jiang et al., 2020). With the development of sampling techniques and the possibility to culture organisms from deep-sea even in conventional standard microbiological laboratories, deep-sea-derived fungi have recently received a wide concern as a new area for bioprospecting (Jin et al., 2016; Pang et al., 2020). As part of our ongoing research for bioactive secondary metabolites from deep-sea-derived fungi (Chen et al., 2016; Wang et al., 2016; Pang et al., 2021), the fungus *Penicillium* sp. SCSIO06868 was studied. Three new tetramic acid derivatives (**1**–**3**) and a new polyketide (**4**) along with eight known compounds (**5**–**12**) (Figure 1) were isolated from the deep-sea-derived fungus *Penicillium* sp. SCSIO06868, which was cultured on a liquid medium. The coronavirus disease 2019 (COVID-19) pandemic has left a mark in more than 180 countries, with more than 2.0 billion cases worldwide and over 4.4 million deaths in total (until August 2021). The COVID-19 is an infectious disease caused by a novel strain of coronavirus [severe acute respiratory syndrome coronavirus 2 (SARS-CoV-2)] (Zehra et al., 2020; Shamsi et al., 2021). SARS-CoV-2 main proteinase (M^Pro^), a key protease of CoV-2, mediates viral replication and transcription. SARS-CoV-2 M^Pro^ has emerged as an attractive target for SARS-CoV-2 drug design and development (Sabbah et al., 2021). All isolated compounds (**1**–**12**) were tested for their antiviral activities against SARS-CoV-2 M^pro^ *in vitro*. Molecular docking research was performed to mimic the interactions between the bioactive compound and SARS-CoV-2 M^pro^. Herein, we described the isolation, structure elucidation, and bioactivity evaluation of the 12 compounds.

## Materials and Methods

### General Experimental Procedures

One-dimensional and two-dimensional (2D) nuclear magnetic resonance (NMR) spectra were measured on a Bruker Avance 700 MHz NMR spectrometer (Fällanden, Switzerland) with Tetramethylsilane as an internal standard. High-resolution electrospray ionization mass spectrometry (HRESIMS) data were recorded on a maXis Q-TOF mass spectrometer in positive ion mode (Bruker, Fällanden, Switzerland). Electronic circular dichroism (ECD) and ultraviolet (UV) spectra were measured with a Chirascan circular dichroism spectrometer (Applied Photophysics). Optical rotations were measured using an MCP-500 polarimeter (Anton, Austria). High-performance liquid chromatography (HPLC) was performed on Hitachi Primaide with YMC ODS SERIES column (YMC-Pack ODS-A, YMC Co. Ltd., Kyoto, 250 × 10 mm I.D., S-5 *μ*m, 12 nm). Column chromatography was carried out on silica gel (200–300 mesh, Jiangyou Silica Gel Development Co., Yantai, China), YMC Gel ODS-A (12 nm, S-50 *μ*m YMC, MA, United States), and Sephadex LH-20 (40–70 *μ*m, Amersham Pharmacia Biotech AB, Uppsala, Sweden). Spots were detected under UV light by heating after spraying with the mixed solvent of saturated vanillin and 5% sulfuric acid in water. The thin layer chromatography plates with silica gel GF254 (0.4–0.5 mm, Qingdao Marine Chemical Factory, Qingdao, China) were used for analysis and preparation.

### Fungal Material

The strain SCSIO06868 was isolated from the deep-sea sediment collected from the Indian Ocean (94°37.377′E; 2°59.853′S; depth 4,762 m). The internal transcribed spacer sequences of SCSIO06868 (494 base pairs, GenBank accession no. MZ277624) have 99% sequence identity to that of *Penicillium citrinum* DUCC5728 (GenBank accession no. 582768). Then, it was designated as a member of *Penicillium* sp. and named as *Penicillium* sp. SCSIO06868. The strain SCSIO06868 was stored on methylene blue agar (malt extract 15 g, agar 16 g, sea salt 10 g, water 1 L, pH 7.4–7.8) slants at 4°C and deposited at Key Laboratory of Tropical Marine Bio-resources and Ecology, Chinese Academy of Sciences.

### Fermentation and Extraction

The mass fermentation of this fungus was carried out in 1-L Erlenmeyer flasks. The fungus was inoculated in a liquid medium (2% maltose, 2% mannitol, 1% monosodium glutamate, 1% glucose, 0.3% yeast extract, 0.05% monopotassium phosphate, 0.03% MgSO_4_⋅7H_2_O, and 300-ml tap water/flask, 93 flasks, 28 L total) at 25°C under static condition for 35 days. After 35 days, the fermentation was soaked in ethyl acetate (500 ml/flask), and the mycelia were cut into small pieces and sonicated for 20 min. The ethyl acetate solution was concentrated under reduced pressure to gain a brown crude extract (59.0 g).

### Isolation and Purification

The crude extract was subjected to silica gel column chromatography, which was eluted with dichloromethane and methanol (MeOH) mixed solvent in a step gradient (100:0–5:1, *v/v*) and separated into seven fractions (Fr-1–Fr-7). Fr-1 (3.2 g) was applied to Sephadex LH-20 column eluted with MeOH, reversed-phase C_18_ medium pressure liquid chromatography (MPLC) eluted with MeOH/water (H_2_O) (10:90–100:0, *v/v*), and semipreparative HPLC [72% CH_3_OH/H_2_O with 0.3‰ trifluoroacetic acid (TFA), 2 ml/min] to afford compounds **7** (2.2 mg, *t*_R_ = 20.2 min) and **8** (18.4 mg, *t*_R_ = 22.2 min). Fr-2 (6.3 g) was subjected to Sephadex LH-20 column eluted with MeOH, reversed-phase C_18_ MPLC eluted with MeOH/H_2_O (10:90–100:0, *v/v*), and semipreparative HPLC (2 ml/min) to gain compounds **3** (4.0 mg, 54% CH_3_CN/H_2_O, *t*_R_ = 31.0 min), **5** (4.5 mg, 35% CH_3_CN/H_2_O with 0.3‰ TFA, *t*_R_ = 33.6 min), **6** (7.6 mg, 35% CH_3_CN/H_2_O with 0.3‰ TFA, *t*_R_ = 35.0 min), and **10** (6.4 mg, 33% CH_3_CN/H_2_O, *t*_R_ = 22.0 min). Fr-3 (4.6 g) was purified with Sephadex LH-20 column eluted with MeOH, reversed-phase C_18_ MPLC eluted with MeOH/H_2_O (10:90–100:0, *v/v*), and semipreparative HPLC (2 ml/min) to obtain compounds **4** (2.9 mg, 72% CH_3_OH/H_2_O with 0.3‰ TFA, *t*_R_ = 8.2 min) and **9** (42.2 mg, 70% CH_3_OH/H_2_O with 0.3‰ TFA, *t*_R_ = 14.6 min). Fr-4 (3.7 g) was purified with Sephadex LH-20 column eluted with MeOH, reversed-phase C_18_ MPLC eluted with MeOH/H_2_O (10:90–100:0, *v/v*), and semipreparative HPLC (75% CH_3_CN/H_2_O with 0.3‰ TFA, 2 ml/min) to yield compound **1** (30.5 mg, *t*_R_ = 12.2 min). Fr-5 (1.3 g) was applied to Sephadex LH-20 column eluted with MeOH, reversed-phase C-18 MPLC eluted with MeOH/H_2_O (10:90–100:0, *v/v*), and semipreparative HPLC (2 ml/min) to get compounds **11** (76.5 mg, 40% CH_3_CN/H_2_O with 0.3‰ TFA, *t*_R_ = 11.4 min) and **12** (60% CH_3_CN/H_2_O, *t*_R_ = 15.2 min, 6.0 mg). Fr-6 (1.6 g) was subjected to Sephadex LH-20 column eluted with MeOH, reversed-phase C_18_ MPLC eluted with MeOH/H_2_O (10:90–100:0, *v/v*), and semipreparative HPLC (75% CH_3_CN/H_2_O with 0.3‰ TFA, 2 ml/min) to obtain compound **2** (2.5 mg, *t*_R_ = 12.8 min).

*Penicillenol G1* (**1**): Pale white solid; ${\left[\alpha \right]}_{\text{D}}^{25}$ –156.6 (*c* 0.10, MeOH); UV (MeOH) λ_*max*_ (log ε) 219 (3.21), and 278 (2.79) nm; ECD (1.06 mM, MeOH) λ_*max*_ (Δε) 209 (+ 5.26), 227 (–5.27), and 281 (−4.38) nm; ^1^H and ^13^C NMR data (Table 1); HRESIMS *m/z* 284.1866 [M + H]^+^ (calcd for C_1__5_H_2__6_NO_4_, 284.1856).

**TABLE 1:** ^1^H NMR (700 MHz) and ^13^C NMR (175 MHz) data for compounds **1**–**3** in CD_3_OD.

No.	1		2		3	
	*δ*_C_, type	*δ*_H_ (*J* in Hz)	*δ*_C_, type	*δ*_H_ (*J* in Hz)	*δ*_C_, type	*δ*_H_ (*J* in Hz)
2	178.0 C		177.7 C		175.4 C	
3	102.3 C		102.8 C		102.8 C	
4	196.2 C		196.0 C		195.1 C	
5	69.0 C	3.73, brs	68.1 CH	3.94, brs	70.0 CH	3.78, brs
6	68.0 C	4.10, qd, 6.3, 2.8	68.7 CH	4.08, qd, 6.3, 3.5	59.3 CH_2_	3.94, qd, 12.6, 2.8
7	20.4 CH_3_	1.29, d, 7.0	17.0 CH_3_	1.10, d, 6.3		
8	193.8 C		194.6 C		192.1 C	
9	37.5 CH	3.58–3.67, m	37.9 CH	3.59–3.67, m	37.2 CH	3.62–3.72, m
10	34.8 CH_2_	1.66–1.74, m	34.9 CH_2_	1.65–1.72, m	34.9 CH_2_	1.68–1.75, m
		1.42–1.50, m		1.40–1.47, m		1.43–1.51, m
11	28.3 CH_2_	1.23–1.35, m	28.4 CH_2_	1.21–1.34, m	28.3 CH_2_	1.23–1.37, m
12	30.3 CH_2_	1.23–1.35, m	30.3 CH_2_	1.21–1.34, m	30.3 CH_2_	1.23–1.37, m
13	23.6 CH_2_	1.23–1.35, m	23.6 CH_2_	1.21–1.34, m	23.6 CH_2_	1.23–1.37, m
14	32.8 CH_2_	1.23–1.35, m	32.9 CH_2_	1.21–1.34, m	32.9 CH_2_	1.23–1.37, m
15	14.4 CH_3_	0.89, t, 7.7	14.4 CH_3_	0.88, t, 7.0	14.4 CH_3_	0.91, t, 6.3
16	17.3 CH_3_	1.16, d, 6.3	17.4 CH_3_	1.14, d, 6.3	17.4 CH_3_	1.17, d, 6.3
17					26.8 CH_3_	3.03, s

*Penicillenol G2* (**2**): Yellowish oil; ${\left[\alpha \right]}_{\text{D}}^{25}$ + 46.0 (*c* 0.10, MeOH); UV (MeOH) λ_*max*_ (log ε) 242 (2.87) and 279 (3.12) nm; ECD (1.06 mM, MeOH) λ_*max*_ (Δε) 213 (–3.08), 238 (+ 2.50), and 271 (2.89) nm; ^1^H and ^13^C NMR data (Table 1); HRESIMS *m/z* 284.1862 [M + H]^+^ (calcd for C_1__5_H_2__6_NO_4_, 284.1856).

*Penicillenol H* (**3**): Yellow oil; ${\left[\alpha \right]}_{\text{D}}^{25}$ –21.0 (*c* 0.10, MeOH); UV (MeOH) λ_*max*_ (log ε) 227 (2.81) and 285 (3.04) nm; ECD (0.71 mM, MeOH) λ_*max*_ (Δε) 213 (2.87), 231 (–2.76), and 289 (–1.27) nm; ^1^H and ^13^C NMR data (Table 1); HRESIMS *m/z* 284.1858 [M + H]^+^ (calcd for C_1__5_H_2__6_NO_4_, 284.1856).

*Coniochaetone N* (**4**): Yellow solid powder; ${\left[\alpha \right]}_{\text{D}}^{25}$ + 46.4 (*c* 0.10, MeOH); UV (MeOH) λ_*max*_ (log ε) 203 (4.11), 226 (4.15), 243 (4.22), and 342 (3.56) nm; ^1^H and ^13^C NMR data (Table 2); HRESIMS *m/z* 285.0373 [M + Na]^+^ (calcd for C_1__3_H_1__0_NaO_6_, 285.0370).

**TABLE 2:** ^1^H NMR (700 MHz) and ^13^C NMR (175 MHz) data for compound **4** in CD_3_OD.

No.	4	
	*δ*_C_, type	*δ*_H_ (*J* in Hz)
1	71.5 CH	5.30, d, 7.0
2	31.4 CH_2_	2.45–2.53, m
		2.00, brt, 11.2
3	30.7 CH_2_	3.19, dt, 17.5, 7.7
		2.89, ddd, 18.2, 9.1, 2.8
4	176.1 C	
6	158.6 C	
7	109.7 CH	7.54, brs
8	138.2 C	
9	113.1 CH	7.32, brs
10	162.3 C	
11	114.3 C	
12	182.2 C	
13	122.9 C	
14	167.9 C	

*X-Ray crystallographic analysis of penicillenol G1* (**1**): Moiety formula: C_1__5_H_2__5_NO_4_ (*M* = 283.36 g/mol), colorless needle, crystal size = 0.6 × 0.03 × 0.03 mm^3^, trigonal, space group C2; unit cell dimensions: *a* = 20.4710(5) Å, *b* = 4.85100(10) Å, *c* = 33.3706(10) Å, *V* = 3154.94(15) Å^3^, *Z* = 8, *ρ*_*calcd*_ = 1.193 g cm^–3^, *T* = 101(2) K, μ(Cu Kα) = 0.698 mm^–1^. A total of 31,816 reflections were measured with 6,225 independent reflections (*R*_*int*_ = 0.0496, *R*_*sigma*_ = 0.0341). Final *R* indices [I > 2σ (*I*)]: *R*_1_ = 0.0348, *wR*_2_ = 0.0872. Final *R* indexes [all date]: *R*_1_ = 0.0407, *wR*_2_ = 0.0896, Flack parameter = 0.07(8). Largest diff. peak and hole = 0.18 and –0.20 eÅ^–3^.

### Molecular Docking Research

The molecular docking was conducted by AutoDockTools (Version 1.5.6) (Morris et al., 2008). The crystal structure of SARS-CoV-2 main protease (PDB ID: 6LU7) was retrieved from the Protein DataBank^1^ (Jin et al., 2020). The structures were generated in ChemBio3D Ultra 14.0 (ChemBioOffice version 14.0), followed by an MM2 calculation to minimize the conformation energy. The original ligand and crystal water were removed before the docking calculation. The hydrogens were added to the structure of 6LU7, and Kollman united partial charges were assigned. A Lamarckian genetic algorithm was applied as a default search algorithm and set the grid box within the size of 46 × 44 × 46 Å, with the spacing of 0.375 Å. During the docking, the default parameters were used if it was not mentioned. The docking pose that had the lowest binding energy was represented as the most favorable binding conformation.

### Antibacterial Activity Assay

All compounds (**1**–**12**) were tested for antibacterial activities against five pathogenic bacteria using the method of agar filter paper diffusion. Compounds that had inhibition zone were evaluated in 96-well plates using a modification of the broth microdilution method (Pang et al., 2018). Ampicillin and gentamicin were used as a positive control for Gram-positive and Gram-negative bacteria, respectively.

### Antiviral Activity Assay

The antiviral activities of all compounds (**1**–**12**) against SARS-CoV-2 M^pro^ were evaluated through the method mentioned in the previous report (Li et al., 2020). Hydroxychloroquine showed potent inhibitory activity against SARS-CoV-2 M^pro^ with *Ki* = 0.36 *μ*m and was used as a positive control.

## Results and Discussion

### Structural Elucidation

**FIGURE 2:**
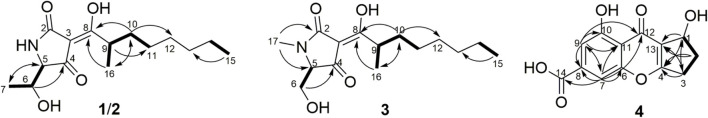
Key ^1^H-^1^H COSY (**—**) and HMBC (→) correlations of compounds **1**–**4**.

**FIGURE 3:**
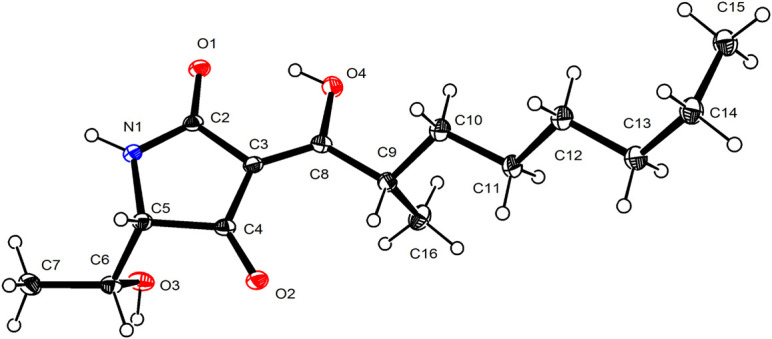
ORTEP drawing of compound **1**.

**FIGURE 4:**
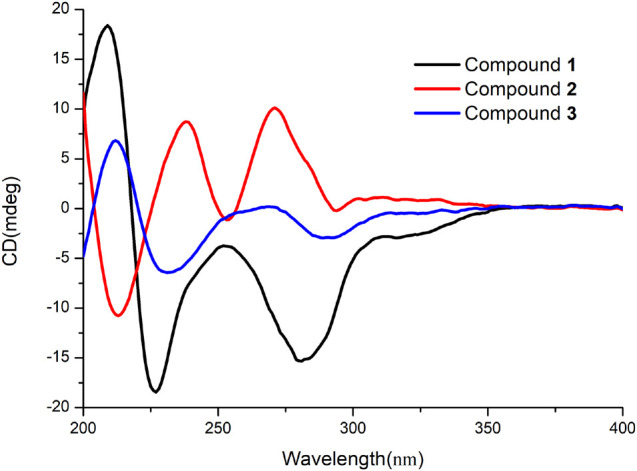
Experimental ECD curves of compounds **1**–**3**.

Penicillenol G1 (**1**) possessed the elemental composition of C_1__5_H_2__5_NO_4_ with 4 degrees of unsaturation as established by its ^13^C NMR data and a protonated molecule at *m*/*z* 284.1866 in the HRESIMS spectrum. Its 1D NMR data (Table 1) displayed three methyls [*δ*_C/H_ 20.4/1.29 (d, *J* = 7.0 Hz, CH_3_-7), 17.3/1.16 (d, *J* = 6.3 Hz, CH_3_-16), and 14.4/0.89 (t, *J* = 7.7 Hz, CH_3_-15)], three sp^3^ methines [(*δ*_C/H_ 69.0/3.73 (brs, CH-5), 68.0/4.10 (dq, *J* = 6.3, 2.8 Hz, CH-6), and 37.5/3.58–3.67 (m, CH-9)], four sp^3^ methenes (*δ*_C/H_ 28.3–34.8/1.23–1.74), and four sp^2^ non-protonated carbons (*δ*_C_ 178.0 C-2, 102.3 C-3, 196.2 C-4, and 193.8 C-8). The ^1^H-^1^H COSY correlations (Figure 2) of H_3_-16/H-9/H_2_-10/H_2_-11 and H_3_-15/H_2_-14, along with four overlapping sp^3^ methenes in the ^1^H NMR, indicated the presence of a 2-isooctyl group. The ^1^H-^1^H COSY correlations of H_3_-7/H-6/H-5 verified that there was a 1-hydroxyethyl group directly connected to C-5. Comparison of the NMR data of **1** with those of penicillenol A_1_ (**5**) (Lin et al., 2008; Yoda et al., 2010) showed that they only differed by an absence of the singlet methyl in **1**. The planar structure of **1** was further confirmed by its heteronuclear multiple bond correlation (HMBC) correlations (Figure 2) of H-6 to C-4, H_3_-7 to C-5, H_2_-10 to C-8 and C-12, and H_3_-16 to C-8 and C-10. The configuration of a double bond at C-3 was determined as *Z* based on that the chemical shift of acylamino (*δ*_C_ 178.0, C-2) was in a lower field than those of normal ones, which caused by the hydrogen bond between the oxygen atom at C-2 and hydroxy at C-8 (Aoki et al., 2000). Thus, the ECD of **1** (Figure 3) displayed a positive Cotton effect at 209 nm (Δ*ε* = + 18.39), a negative Cotton effect at 227 nm (Δ*ε* = –15.33), and a negative Cotton effect at 281 nm (Δ*ε* = –4.38), and the trend of which was consistent with that of **5**. Thus, the absolute configuration of C-5 in **1** was determined as *S*. Mosher’s method was tried to confirm the absolute configuration of C-6 but failed. Fortunately, the single crystal of **1** was obtained, and the absolute configuration of **1** was established as 5*S*, 6*R*, 9*S* by analyzing the X-ray crystallographic data (Figure 4). Compound **1** was named as penicillenol G1.

The molecular formula of penicillenol G2 (**2**), which was the same as **1**, was established as C_1__5_H_2__5_NO_4_ by its NMR data (Table 1) and a protonated molecule at *m*/*z* 284.1862 in the HRESIMS. Its NMR data were nearly the same as those of **1**, and only the chemical shifts of C-5 (*δ*_C/H_ 68.1/3.94, brs), C-6 [*δ*_C/H_ 68.7/4.08 (qd, *J* = 6.3, 3.5 Hz)], and C-7 [*δ*_C/H_ 17.0/1.10 (d, *J* = 6.3 Hz)] in **2** have some small differences. Compound **2** had the same planar structure with **1**, which was confirmed by the ^1^H-^1^H COSY and HMBC spectra (Figure 2). Thereby, the differences mentioned earlier might be caused by their different configurations. The ECD spectrum of **2** showed a negative Cotton effect at 213 nm (Δ*ε* = –3.08), a positive Cotton effect at 238 nm (Δ*ε* = + 2.5), and a positive Cotton effect at 271 nm (Δ*ε* = + 2.89), which exhibited a reverse trend with **1** and **5**, but was consistent with penicillenol A2 (**6**) (Lin et al., 2008; Sengoku et al., 2012). Therefore, the absolute configuration of C-5 in **2** was determined as *R*. Some synthetic chemists demonstrated that the natural penicillenol A1 (**5**) (Yoda et al., 2010), penicillenol A2 (**6**) (Sengoku et al., 2012), and penicillenol C1 (**7**) (Kempf et al., 2013) were all have the same absolute configurations of 6*R* and 9*S*, and the absolute configuration of compound **1** was also determined as 6*R* and 9*S* by the X-ray diffraction study. Thus, considering the same biosynthetic pathway (Yin et al., 2019) and comparison of the spectroscopic data with **1**, the absolute configurations of C-6 and C-9 in **2** were deduced as 6*R*, 9*S*.

Penicillenol H (**3**) was obtained as a yellow oil. The ^13^C NMR data and a protonated molecule at *m*/*z* 284.1858 in the HRESIMS of **3** suggested that its molecular formula was C_1__5_H_2__5_NO_4_ with 4 degrees of unsaturation. The NMR data (Table 1) of **3** were similar to those of **1**, except that the 1-hydroxyethyl group in **1** was displaced by oxygenated methylene (*δ*_C/H_ 59.3/3.94, qd, *J* = 12.6, 2.8 Hz, CH_2_-6) and an *N*-methyl group (*δ*_C/H_ 26.8/3.03, s, CH_3_-17) was added in **3**. The extinction was determined by its ^1^H-^1^H COSY cross-peak of H_2_-6/H-5 and HMBC correlations of H_2_-6 to C-4 and H_3_-17 to C-2 and C-5. The planar structure of **3** was further established by its 2D NMR (Figure 2). The configuration of a double bond at C-3 was designated as *Z* by the chemical shift of C-2 (*δ*_C_ 178.0) (Aoki et al., 2000). The absolute configuration of C-5 and C-9 in **3** were determined as *S* and *R* same as compound **1** by their similar ECD spectrum (Figure 3), of which **3** showed a positive Cotton effect at 213 nm (Δ*ε* = + 2.87), two negative Cotton effects at 231 nm (Δ*ε* = – 2.76), and 289 nm (Δ*ε* = – 1.27), respectively. Compound **3** was named penicillenol H.

The molecular formula of compound **4** was C_1__3_H_1__0_O_8_, established by its ^13^C NMR data (Table 2) and a sodium adduct ion peak at *m*/*z* 285.0373 in the HRESIMS spectrum. Its ^1^H NMR data were simple and showed two aromatic protons (*δ*_H_ 7.54, brs, CH-7; 7.32, brs, CH-9) at meta-position, an oxygenated methine (*δ*_H_ 5.30, d, *J* = 7.0 Hz, CH-1), and two sp^3^ methylenes (*δ*_H_ 2.45–2.53, m, 2.00, brt, *J* = 11.2 Hz, CH_2_-2; 3.19, dt, *J* = 17.5, 7.7 Hz, 2.89, ddd, *J* = 18.2, 9.1, 2.8 Hz, CH_2_-3). Besides the corresponding carbons (*δ*_C_ 109.7, CH-7; 113.1, CH-9; 71.5, CH-1; 34.4, CH_2_-2; 30.7, CH_2_-3), there were eight sp^2^ non-protonated carbons in its ^13^C NMR, which indicated the presences of an *α*,*β*-unsaturated ketone (*δ*_C_ 182.2, C-12; 122.9, C-13; 176.1, C-4) and a carboxyl (*δ*_C_, 167.9, C-14). Its NMR data were very similar to those of coniochaetone L (Guo et al., 2019), except that the methoxy group at C-1 in coniochaetone L was replaced by hydrogen in **4**. The speculation was further confirmed by its HMBC and ^1^H-^1^H COSY spectra (Figure 2). The positive specific rotation of **4** (${\left[\alpha \right]}_{\text{D}}^{25}$ + 46.4, MeOH) that was consistent with that of coniochaetone L (${\left[\alpha \right]}_{\text{D}}^{25}$ + 25.3, MeOH) suggested that the configuration of C-1 was *R*. Thus, compound **4** was established as *R*-1,8-dihydroxy-9-oxo-1,2,3,9- tetrahydrocyclopenta[b]chromene-6-carboxylic acid and named as coniochaetone N.

In addition, the eight known compounds (**5**–**12**) (Figure 1) were identified as penicillenol A1 (**5**) (Lin et al., 2008; Yoda et al., 2010), penicillenol A2 (**6**) (Lin et al., 2008; Sengoku et al., 2012), penicillenol C1 (**7**) (Lin et al., 2008; Kempf et al., 2013), penicillenol C2 (**8**) Lin et al., 2008), scalusamide C (**9**) (Tsuda et al., 2005), (*E*)-7-(3-methyl-4-oxo-6,7,8,8a-tetrahydro-4*H*-pyrrolo[2,1-b][1,3]oxazin-2-yl)hept-2-enoic acid (**10**) (Lai et al., 2013), terretrione D (**11**) (Shaala and Youssef, 2015), and (2*R*)-2,3-dihydro-7-hydroxy-6,8-dimethyl-2-[(*E*)-prop-1-enyl] chromen-4-one (**12**) (Li et al., 2007; Zhang et al., 2019) by comparison of their physical and spectroscopic data with those in the literature.

### Bioassays of Compounds

**FIGURE 5:**
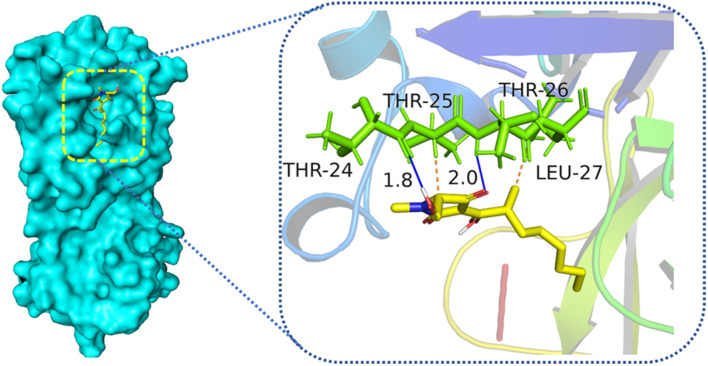
Low-energy binding conformations the complex between compound **3** and SARS-CoV-2 M^pro^ by virtual docking. Blue solid line means hydrogen bonds, and orange dotted lines mean hydrophobic interactions.

All isolated compounds (**1**–**12**) were tested for their antiviral activities against SARS-CoV-2 M^pro^ *in vitro* through the method mentioned in the reported literature (Li et al., 2020). Compound **3** showed weak inhibitory activity against M^pro^ enzyme, which was responsible for the COVID-19 pandemic. When treated with 50 *μ*m of **3**, the relative enzyme activity of SARS-CoV-2 M^pro^ was 46.64%, and that of the positive control hydroxychloroquine was 5.28% with the same concentration. To better understand the interactions between compounds and SARS-CoV-2 M^pro^, molecular docking research was performed to mimic the interactions between compound **3** and M^pro^ enzyme of SARS-CoV-2 (PDB ID: 6LU7) by utilizing the AutoDockTools. Molecular docking results demonstrated that compound **3** could interact with the SARS-CoV-2 M^pro^ enzyme at the entrance of the catalytic pocket, with the calculated binding affinities of –4.98 kcal/mol. The 2D binding model for **3** (Figure 5) showed two hydrogen bonds and two intermolecular hydrophobic interactions. Two hydrogen bonds were formed between the carbonyl group at C-4 and Thr-26, as well as between the hydroxyl group at C-6 and Thr-24. The lengths of the two hydrogen bonds were 2.0 and 1.8 Å, respectively. These results suggested that compound **3** could insert into the active site of the enzyme and bind tightly to the catalytic amino acid residues by different types of interactions to inhibit SARS-CoV-2 M^pro^.

Compounds **1**–**12** were evaluated their antibacterial activities against five pathogenic bacteria *Escherichia coli* (ATCC 25922), *Enterococcus faecalis* (ATCC 29212), *Klebsiella pneumonia* (ATCC 13883), *Staphyloccocus aureus* (ATCC 29213), and methicillin-resistant *S. aureus* (MRSA). Compounds **1**, **3**, **5,** and **6** with 50 *μ*g/disc showed inhibition zones against *S. aureus*. Compounds **1**, **3**, and **5** with 50 *μ*g/disc showed an inhibition zone against MRSA (Supplementary Figure 1). Furthermore, their minimum inhibitory concentrations (MICs) were tested, and the results are shown in Table 3. Compound **3** displayed potent inhibitory activities against *S. aureus* and MRSA with MIC values of both 2.5 μg/ml. Ampicillin was used as a positive control against *S. aureus* and MRSA with MIC values of 1.56 and 0.39 μg/ml, respectively.

**TABLE 3:** MIC values (μg/ml) of compounds with antibacterial activities.

Compounds	*S. aureus*	MRSA
1	20	40
3	2.5	2.5
5	40	40
6	80	–
Ampicillin	1.56	0.39

## Conclusion

In summary, we reported the isolation and identification of three new tetramic acid derivatives (**1**–**3**) and a new polyketide (**4**) along with eight known compounds (**5**–**12**) from cultures of the deep-sea-derived fungus *Penicillium* sp. SCSIO06868. The absolute configurations of new compounds were established by X-ray crystallography analysis and comparison of the experimental and reported ECD value or specific optical rotation. Compound **3** displayed potent inhibitory activities against *S. aureus* and MRSA with MIC values of both 2.5 *μ*g/ml. Compound **3** showed weak inhibitory activity against M^pro^ enzyme of SARS-CoV-2, which was responsible for the COVID-19 pandemic. Molecular docking was performed to mimic the interactions between compound **3** and SARS-CoV-2 M^pro^. The molecular docking results indicated that compound **3** could be inserted into the active site of the enzyme and bind tightly to the catalytic amino acid residues by different types of interactions to inhibit SARS-CoV-2 M^pro^.

## Data Availability Statement

The datasets presented in this study can be found in online repositories. The names of the repository/repositories and accession number (s) can be found in the article/Supplementary Material.

## Author Contributions

XP, JW, and YL contributed to conception and design of the study. XP performed the experiments, analyzed the data, and wrote the manuscript. XW performed the SARS-CoV-2 M^pro^ inhibition test. XT did the isolation of the fungus. All authors contributed to manuscript revision, review, and approved the submitted version.

## Conflict of Interest

The authors declare that the research was conducted in the absence of any commercial or financial relationships that could be construed as a potential conflict of interest.

## Publisher’s Note

All claims expressed in this article are solely those of the authors and do not necessarily represent those of their affiliated organizations, or those of the publisher, the editors and the reviewers. Any product that may be evaluated in this article, or claim that may be made by its manufacturer, is not guaranteed or endorsed by the publisher.

## Footnotes

1
http://www.rcsb.org/pdb
