# Supplementary material for: New Tetramic Acid Derivatives From the Deep-Sea-Derived Fungus Penicillium sp. SCSIO06868 With SARS-CoV-2 Mpro Inhibitory Activity Evaluation
Source: Front Microbiol. 2021 Sep 27;12:730807. doi: 10.3389/fmicb.2021.730807 (PMC8503681; doi:10.3389/fmicb.2021.730807)
Supplement: Supplementary file 1 [file Data_Sheet_1.PDF]

Supplementary Material

New tetramic acid derivatives from the deep-sea-derived fungus *Penicillium* sp. SCSIO06868 with SARS-CoV-2 M^{pro} inhibitory activity evaluation

Xiaoyan Pang[1,2,3], Weihao Chen[1], Xin Wang[4], Xuefeng Zhou[1,2], Bin Yang[1,2], Xinpeng Tian[1,2], Junfeng Wang[1,2*], Shihai Xu[3*], Yonghong Liu[1,2*]

[1]CAS Key Laboratory of Tropical Marine Bio-Resources and Ecology, Guangdong Key Laboratory of Marine Materia Medica, Innovation Academy of South China Sea Ecology and Environmental Engineering, South China Sea Institute of Oceanology, Chinese Academy of Sciences, Guangzhou, 510301, China;

[2] Sanya Institute of Oceanology, SCSIO, Yazhou Scientific Bay, Sanya 572000, China;

[3]College of Chemistry and Materials Science, Jinan University, Guangzhou 510632, China;

[4]Center for Innovative Marine Drug Screening & Evaluation, School of Medicine and Pharmacy, Ocean University of China, 266100 Qingdao, China;

*** Correspondence:**
wangjunfeng@scsio.ac.cn; Tel: +86-020-8902-3174 (J.W.);

txush@jnu.edu.cn; Tel: +86-020-85220223 (S.X.);

yonghongliu@scsio.ac.cn; Tel: +86-020-8902-3244 (Y.L.).

Keywords: deep-sea-derived fungus, *Penicillium* sp., secondary metabolites, antibacterial, antiviral

1. Supplementary Data

1.1 The NMR and mass data of known compounds isolated from the deep-sea-derived fungus *Penicillium* sp. SCSIO06868

Penicillenol A1 (**5**)，^{1}H NMR (700 MHz, CD_3OD) δ_H: 3.72 (brs, H-5), 4.18 (dq, *J*=7.0, 3.5 Hz, H-6), 1.32 (d, *J*=6.3 Hz, H-7), 3.61–3.68 (m, H-9), 1.65–1.72 (m, H-10a), 1.43–1.50 (m, H-10b), 1.21–1.32 (m, H_2-11, H_2-12, H_2-13, H_2-14), 0.89 (t, *J*=7.0 Hz, H_3-15), 1.16 (d, *J*=6.3, H_3-16), 3.08 (s, H_3-17); ^{13}C NMR (175 MHz, CD_3OD) δ_C: 175.7 (C-2), 102.4 (C-3), 195.2 (C-4), 68.4 (CH-5), 72.9 (CH-6), 20.5 (CH_3-7), 191.8 (C-8), 37.1 (CH-9), 34.8 (CH_2-10), 28.3 (CH_2-11), 30.3 (CH_2-12), 23.6 (CH_2-13), 32.8 (CH_2-14), 14.4 (CH_3-15), 17.4 (CH_3-16), 29.6 (CH_3-17). HRESIMS *m/z* 298.2010 $[M+H]^+$, (calcd for $C_{16}H_{28}NO_4$, 298.2013).

Penicillenol A2 (**6**), ^{1}H NMR (700 MHz, CD_3OD) δ_H: 3.83 (brs, H-5), 4.26 (dq, *J*=6.3, 2.1 Hz, H-6), 1.12–1.20 (m, H-7), 3.60-3.75 (m, H-9), 1.67–1.74 (m, H-10a), 1.40–1.50 (m, H-10b), 1.23–1.37 (m, H_2-11, H_2-12, H_2-13, H_2-14), 0.90 (t, *J*=7.0 Hz, H_3-15), 1.12–1.20 (m, H_3-16), 3.06 (s, H_3-17); ^{13}C NMR (175 MHz, CD_3OD) δ_C: 175.3 (C-2), 102.8 (C-3), 194.3 (C-4), 68.1 (CH-5), 72.9 (CH-6), 17.5 (CH_3-7), 192.1 (C-8), 37.3 (CH-9), 34.9 (CH_2-10), 28.4 (CH_2-11), 30.4 (CH_2-12), 23.7 (CH_2-13), 32.9 (CH_2-14), 14.4 (CH_3-15), 17.1 (CH_3-16), 28.2 (CH_3-17). ESIMS *m/z* 298.8 $[M+H]^+$.

Penicillenol C1 (**7**), ^{1}H NMR (700 MHz, CD_3OD) δ_H:3.70 (brs, H-5), 4.20 (dq, *J*=6.3, 2.8 Hz, H-6), 1.28–1.33 (m, H-7), 3.70 (brs, H-9), 1.65–1.73 (m, H-10a), 1.33–1.40 (m, H-10b and H_2-11), 1.97 (d, *J*=6.3, H_2-12), 5.34–5.47 (m, H-13 and H-14), 1.63 (d, *J*=4.9 Hz, H_3-15), 1.15 (d, *J*=6.3 Hz, H_3-16), 3.06 (brs, H_3-17); ^{13}C NMR (175 MHz, CD_3OD) δ_C: 175.2 (C-2), 102.7 (C-3), 195.4 (C-4), 68.3 (CH-5), 72.6 (CH-6), 20.3 (CH_3-7), 191.8 (C-8), 37.2 (CH-9), 34.4 (CH_2-10), 28.4 (CH_2-11), 33.5 (CH_2-12), 132.2 (CH-13), 126.0 (CH-14), 18.1 (CH_3-15), 17.5 (CH_3-16), 29.4 (CH_3-17). ESIMS *m/z* 296.8 $[M+H]^+$.

Penicillenol C2 (**8**), ^{1}H NMR (700 MHz, CD_3OD) δ_H:3.85 (brs, H-5), 4.26 (dq, *J*=6.3, 2.1 Hz, H-6), 1.17 (d, *J*=7.0, H-7), 3.64 (brs, H-9), 1.65–1.74 (m, H-10a), 1.44–1.52 (m, H-10b), 1.25–1.39 (m, H_2-11), 1.92–2.03 (m, H_2-12), 5.35–5.46 (m, H-13 and H-14), 1.63 (d, *J*=6.3 Hz, H_3-15), 1.17 (d, *J*=7.0 Hz, H_3-16), 3.07 (s, H_3-17); ^{13}C NMR (175 MHz, CD_3OD) δ_C: 175.9 (C-2), 102.7 (C-3), 194.3 (C-4), 68.0 (CH-5), 73.0 (CH-6), 17.5 (CH_3-7), 191.5 (C-8), 36.7 (CH-9), 34.3 (CH_2-10), 28.3 (CH_2-11), 33.4 (CH_2-12), 132.0 (CH-13), 126.1 (CH-14), 18.1 (CH_3-15), 17.2 (CH_3-16), 30.3 (CH_3-17). ESIMS *m/z* 296.8 $[M+H]^+$.

Scalusamide C (**9**), ^{1}H NMR (700 MHz, CD_3OD) δ_H: 4.20-4.27 (m, H-2a), 1.85-1.91(m, H-3aa), 2.02-2.08 (m, H-3ab), 1.94-2.0 (m, H_2-4a), 3.42(m, H-5aa), 3.48 (m, H-5ab), 3.54 (m, H-7a), 2.44-2.57 (m, H_2-9a), 1.56 (dq, *J*=14.7, 7.0 Hz, H_2-10a), 1.29-1.34 (m, H_2-11a), 1.94-2.0 (m, H_2-12a), 5.35-5.44 (m, H-13a, 14a), 1.63 (d, J=4.9 Hz, H_3-15a), 3.58 (dd, *J*=11.9, 7.7 Hz, H_2-16a), 1.36 (d, J=7.0 Hz, H_3-17a), 4.20-4.27 (m, H-2b), 1.85-1.91(m, H-3ba), 2.02-2.08 (m, H-3bb), 1.94-2.0 (m, H_2-4b), 3.42(m, H-5ba), 3.48 (m, H-5bb), 3.54 (m, H-7b), 2.44-2.57 (m, H_2-9b), 1.56 (dq, *J*=14.7, 7.0 Hz, H_2-10b), 1.29-1.34 (m, H_2-11b), 1.94-2.0 (m, H_2-12b), 5.35-5.44 (m, H-13b, 14b), 1.63 (d, *J*=4.9 Hz, H_3-15b), 3.56 (dd, *J*=13.3, 5.6 Hz, H_2-16b), 1.39 (d, *J*=7.0 Hz, H_3-17b); ^{13}C NMR (175 MHz, CD_3OD) δ_C: (61.4, CH-2a), (28.2, CH_2-3a), (24.5, CH_2-4a), (48.3, CH_2-5a), (171.6, C-6a), (53.3, CH-7a), (207.2, C-8a), (39.7, CH_2-9a), (23.0, CH_2-10a), (29.0, CH_2-11a), (32.3, CH_2-12a), (130.9, CH_2-13a), (125.2, CH_2-14a), (17.9, CH_3-15a), (66.8, CH_2-16a), (13.8, CH_3-17a), (61.2, CH-2b), (28.1, CH_2-3b), (24.3, CH_2-4b), (48.2, CH_2-5b), (171.1, C-6b), (53.3, CH-7b), (207.2, C-8b), (39.5, CH_2-9b), (23.0, CH_2-10b), (29.0, CH_2-11b), (32.3, CH_2-12b), (130.9, CH-13b), (125.2, CH-14b), (17.9, CH_3-15b), (66.2, CH_2-16b), (13.1, CH_3-17b).ESIMS *m/z* 282.8 $[M+H]^+$.

(*E*)-7-(3-methyl-4-oxo-6,7,8,8a-tetrahydro-4*H*-pyrrolo[2,1-b][1,3]oxazin-2-yl)hept-2-enoic acid (**10**), ^{1}H NMR (700 MHz, DMSO-*d*) δ_H:2.23 (q, *J*=7.0 Hz, H_2-4), 1.56–1.62 (m, H_2-5), 1.49–1.54 (m, H_2-6), 2.38 (tq, *J*=14.7, 7.7

Hz, H_2-7), 6.84 (dt, *J*=15.4, 7.0 Hz, H-8), 5.82 (d, *J*=15.4 Hz, H-9), 1.78 (s, CH_3-11), 5.27 (t, *J*=5.6 Hz, CH-12), 2.27 (dt, *J*=14.7, 7.0 Hz, H-13a), 2.12 (dtd, *J*=12.6, 7.7, 4.9 Hz, H-13b), 2.01 (tt, *J*=14.0, 7.7 Hz, H-14a), 1.88–1.95 (m, H-14b), 3.65 (dt, J=11.2, 7.0 Hz, H-15a), 3.40 (ddd, J=11.2, 7.7, 6.3 Hz, H-15b); ^{13}C NMR (175 MHz, DMSO-*d*) δ_C: 165.5 (C-1), 107.1 (C-2), 165.9 (C-3), 32.5 (CH_2-4), 27.3 (CH_2-5), 28.8 (CH_2-6), 32.7 (CH_2-7), 147.1 (CH-8), 125.0 (CH-9), 172.6 (C-10), 10.2 (CH_3-11), 89.1 (CH-12), 31.1 (CH_2-13), 22.7 (CH_2-14), 45.4 (CH_2-15). ESIMS *m/z* 280.7 $[M+H]^+$, 302.8 $[M+Na]^+$, 581.3 $[2M+Na]^+$.

Terretrione D (**11**) ^{1}H NMR (700 MHz, $CDCl_{3.}$) δ_H: 4.36 (dd, *J*=7.7, 3.5 Hz, H-3), 5.04 (brd, *J*=7.0 Hz, H-6), 1.89 (s, H_3-8), 1.98–2.06 (m, H-1'), 0.75 (d, *J*=7.0 Hz, H_3-2'), 0.72 (d, *J*=7.0 Hz, H_3-3') , 3.02 (qd, *J*=13.3, 8.4 Hz, H_2-1''), 7.18–7.22 (m, H-3'', H-5'' and H-7''), 7.22–7.26 (m, H-4'' and H-6'') ; ^{13}C NMR (175 MHz, $CDCl_{3.}$) δ_C: 171.8 (C-2), 57.7 (CH-3), 174.2 (C-5), 54.8 (CH-6), 172.2 (C-7), 22.6 (CH_3-8), 30.8 (CH-1'), 18.8 (CH_3-2'), 17.7 (CH_3-3'), 39.1 (CH_2-1''), 136.4 (C-2''), 129.4 (CH-3''), 128.6 (CH-4''), 127.1 (CH-5''), 129.4 (CH-6''), 130.2 (CH-H7'').

(2*R*)-2,3-dihydro-7-hydroxy-6,8-dimethyl-2-[(*E*)-prop-1-enyl] chromen-4-one (**12**) ,^{1}H NMR (700 MHz, CD_3OD) δ_H:5.49 (s, H-5), 2.68 (dd, *J*=16.8, 3.5 Hz, H-3a), 2.60 (dd, *J*=16.8, 11.9 Hz, H-3b), 7.43 (s, H-5), 5.91 (dqd, *J*=15.4, 7.0, 0.7 Hz, H-9), 5.72 (ddq, *J*=15.4, 6.3, 1.4 Hz, H-10), 1.76 (brd, *J*=7.0 Hz, H_2-11), 2.16 (brs, 6-CH_3), 2.08 (s, 8-CH_3); ^{13}C NMR (175 MHz, MeOD) δ_C: 79.4 (CH-2), 43.5 (CH_2-3), 194.1 (C-4), 114.7 (C-4a), 126.3 (CH-5), 119.9 (C-6), 161.2 (C-7), 112.5 (C-8), 130.4 (CH-9), 130.5 (CH-10), 17.9 (CH_2-11), 16.3 (6-CH_3), 8.5 (6-CH_3). ESIMS *m/z* 233.5 $[M+H]^+$, 255.6 $[M+Na]^+$, 487.5 $[2M+Na]^+$.

1.2. The ITS sequences data of *Penicillium* sp. SCSIO06868

CCACCCGTGTTGCCCGAACCTATGTTGCCTCGGCGGGCCCCGCGCCCGCCGACGGCCCC

CCTGAACGCTGTCTGAAGTTGCAGTCTGAGACCTATAACGAAATTAGTTAAAACTTTCA

ACAACGGATCTCTTGGTTCCGGCATCGATGAAGAACGCAGCGAAATGCGATAACTAATG

TGAATTGCAGAATTCAGTGAATCATCGAGTCTTTGAACGCACATTGCGCCCTCTGGTATT

CCGGAGGGCATGCCTGTCCGAGCGTCATTGCTGCCCTCAAGCCCGGCTTGTGTGTTGGG

CCCCGTCCCCCCCGCCGGGGGACGGGCCCGAAAGGCAGCGGCGGCACCGCGTCCGGT

CCTCGAGCGTATGGGGCTTCGTCACCCGCTCTAGTAGGCCCGGCCGGCGCCAGCCGACC

CCCAACCTTTAATTATCTCAGGTTGACCTCGGATCAGGTAGGGATACCCGCTGAACTTA

AGCATATCAATAAGCGGAGGAA

2. Supplementary Figures

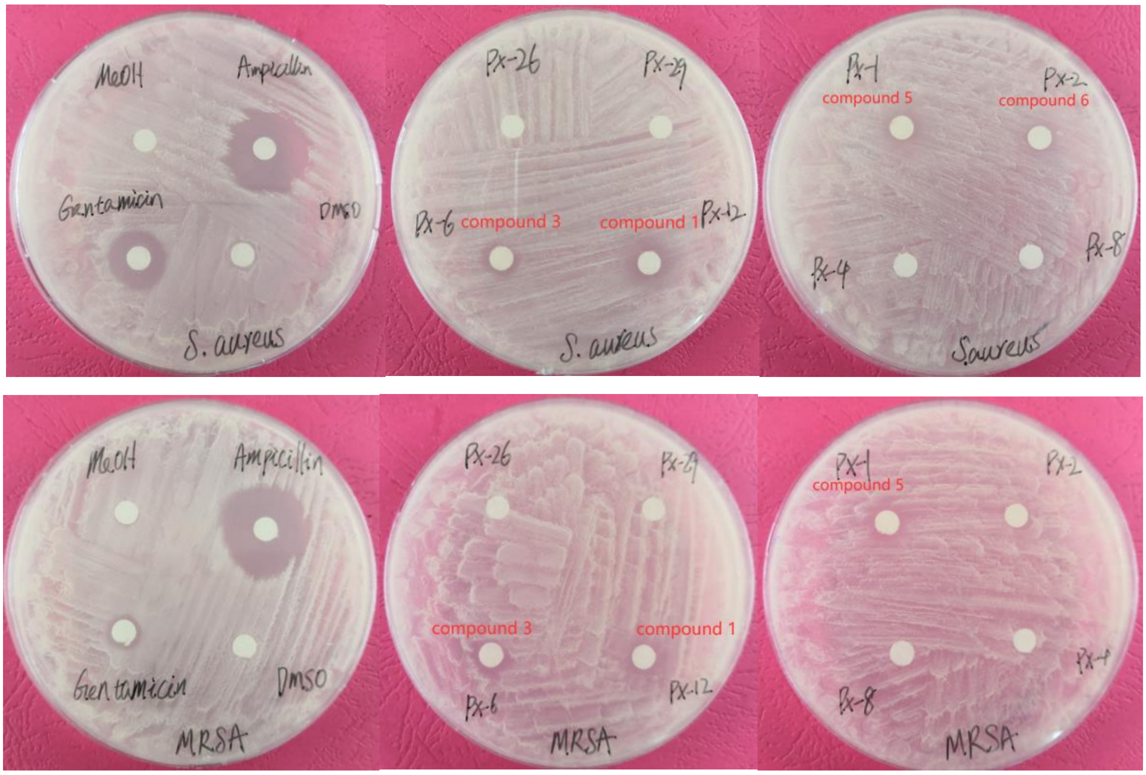

Figure S1. The preliminary screening results of the antibacterial assay.

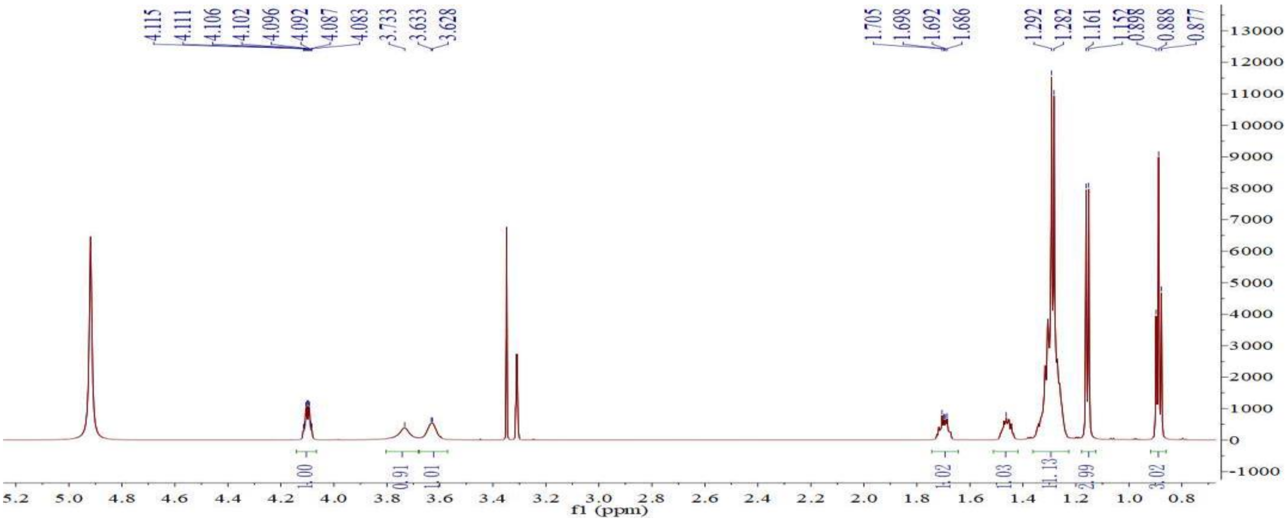

Figure S2. ^{1}H NMR spectrum of **1** in CD_3OD.

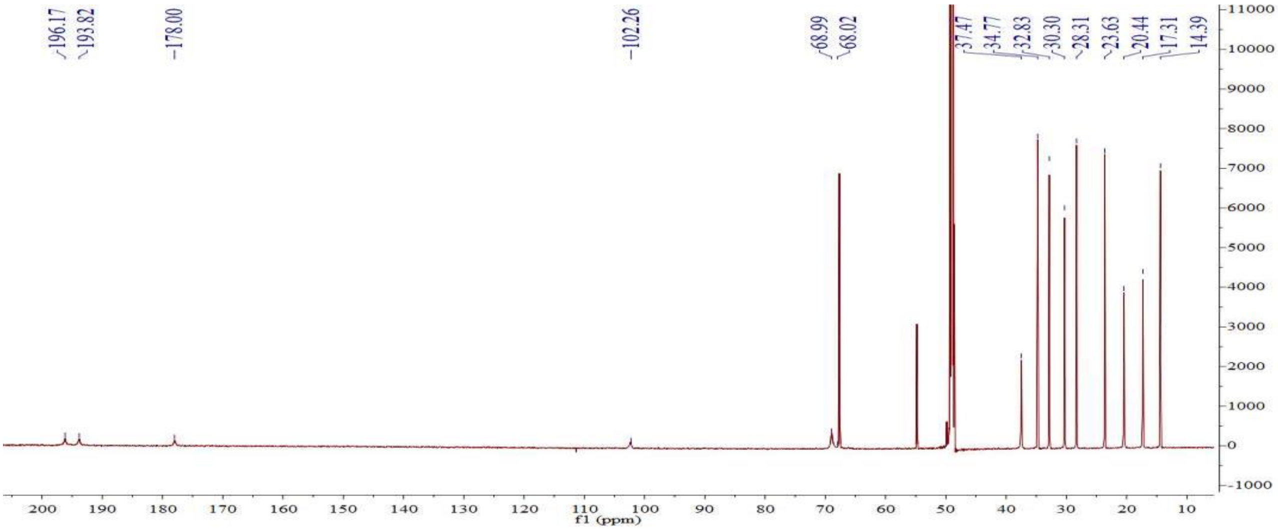

Figure S3. ^{13}C NMR spectrum of **1** in CD_3OD.

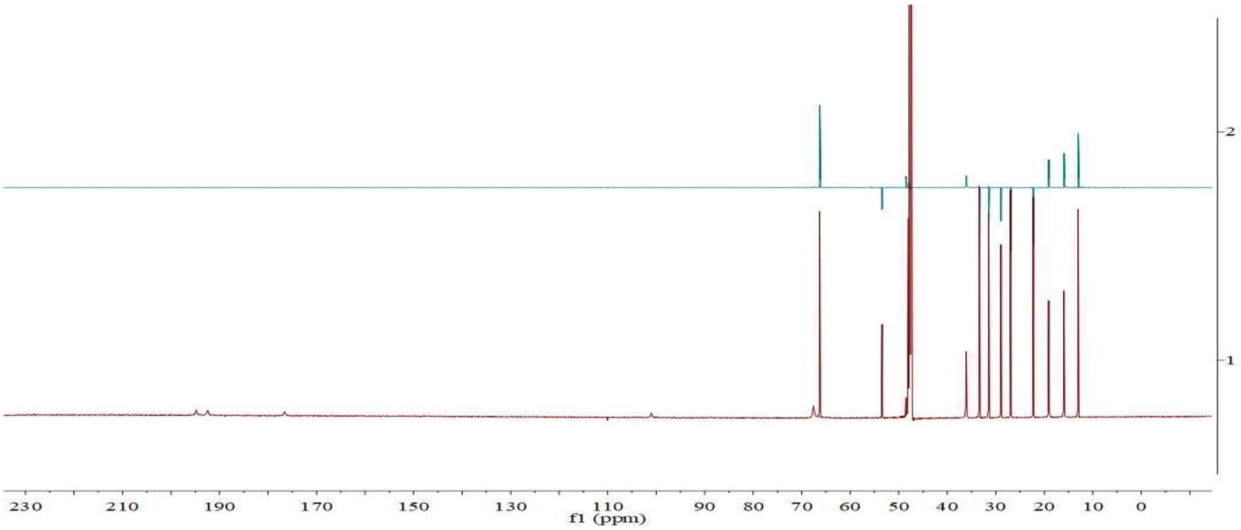

Figure S4. DEPT NMR spectrum of **1** in CD_3OD.

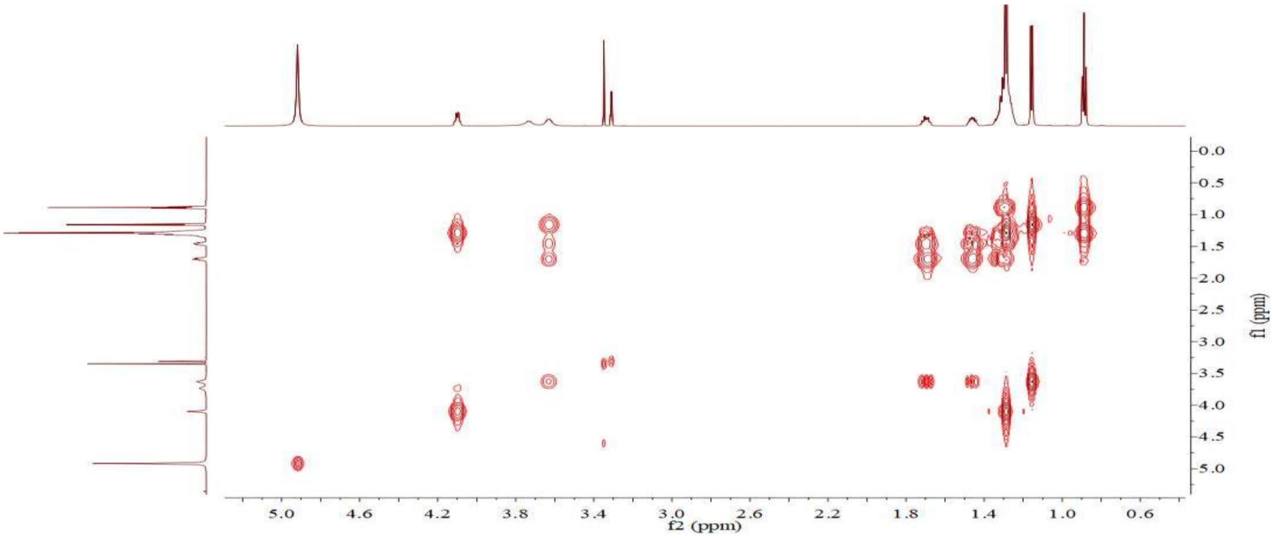

Figure S5. ^{1}H-^{1}H COSY spectrum of **1** in CD_3OD.

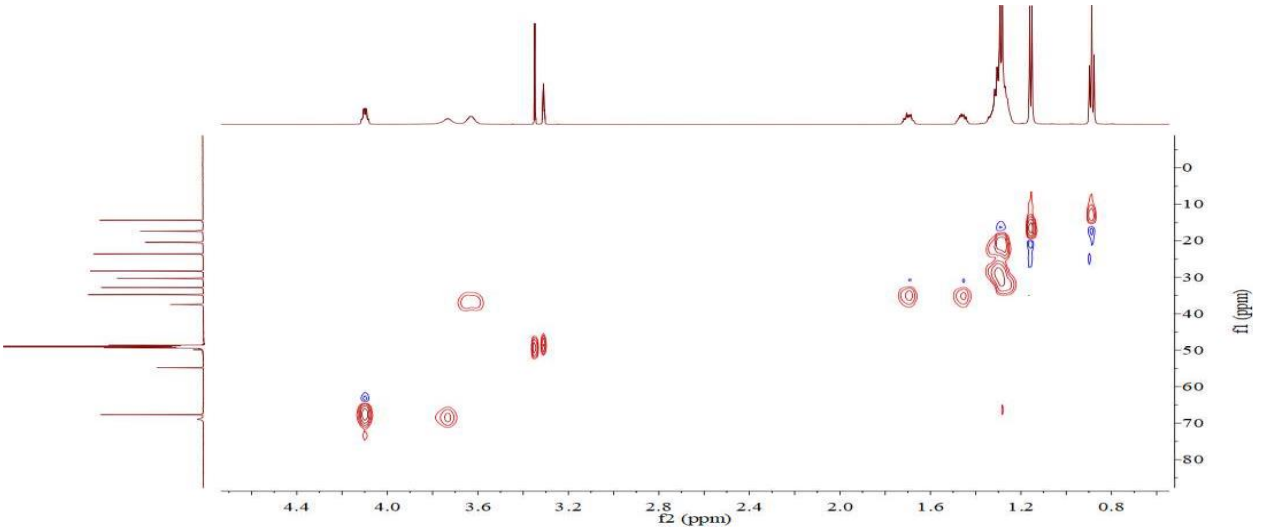

Figure S6. HSQC spectrum of **1** in CD_3OD.

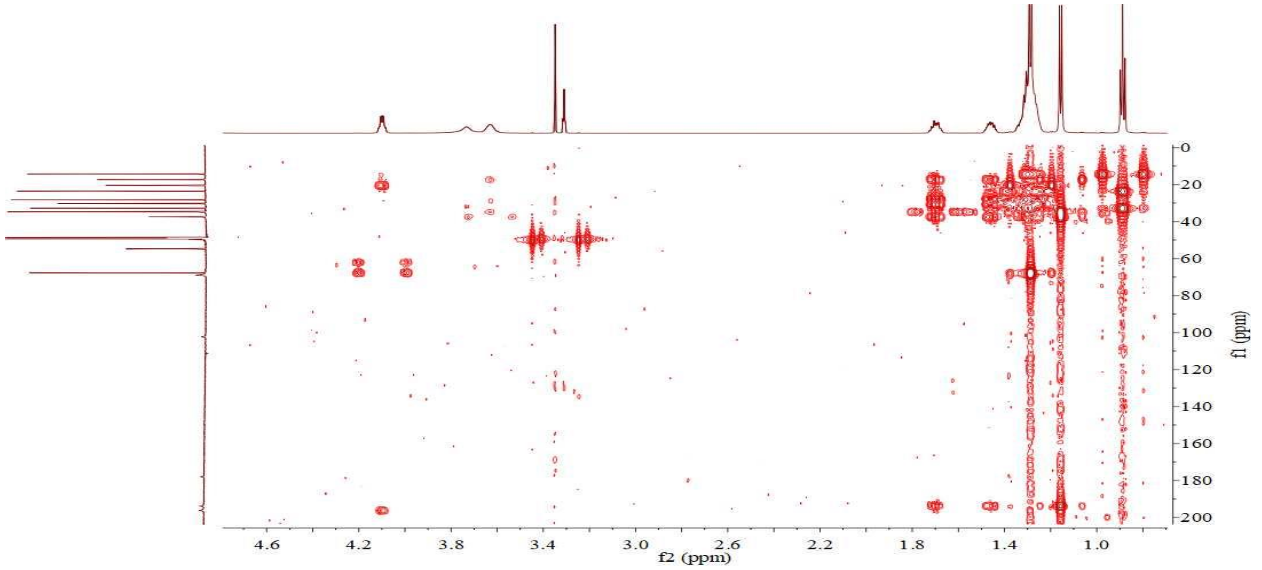

Figure S7. HMBC spectrum of **1** in CD_3OD.

Mass Spectrum SmartFormula Report

Analysis Info

Analysis Name	D:\Data\MS\data\201904\pangxiaoyan_px-12_pos_12_01_6462.d	Acquisition Date	4/11/2019 11:07:27 AM
Method	LC_Direct Infusion_pos_70-500mz.m	Operator	SCSIO
Sample Name	pangxiaoyan_px-12_pos	Instrument	maXis 255552.00029
Comment			

Acquisition Parameter

Source Type	ESI	Ion Polarity	Positive	Set Nebulizer	0.4 Bar
Focus	Active	Set Capillary	4500 V	Set Dry Heater	180 °C
Scan Begin	70 m/z	Set End Plate Offset	-500 V	Set Dry Gas	4.0 l/min
Scan End	1500 m/z	Set Charging Voltage	0 V	Set Divert Valve	Waste
		Set Corona	0 nA	Set APCI Heater	0 °C

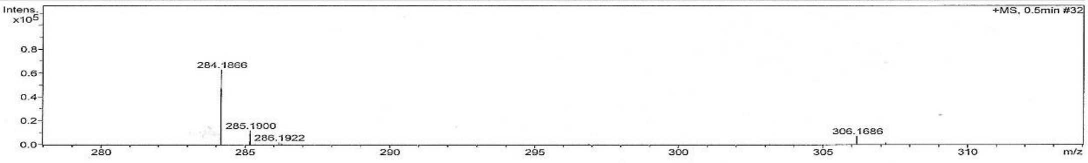

Meas. m/z	#	Ion Formula	Score	m/z	err [ppm]	err [mDa]	mSigma	rdb	e⁻ Conf	N-Rule
284.1866	1	C15H26NO4	100.00	284.1856	-3.6	-1.0	12.4	3.5	even	ok
306.1686	1	C15H25NNaO4	100.00	306.1676	-3.3	-1.0	15.3	3.5	even	ok
567.3637	1	C30H51N2O8	100.00	567.3640	0.5	0.3	20.6	6.5	even	ok
589.3467	1	C30H50N2NaO8	100.00	589.3459	-1.3	-0.8	61.9	6.5	even	ok

pangxiaoyan_px-12_pos_12_01_6462.d
Bruker Compass DataAnalysis 4.1 printed: 4/11/2019 11:11:35 AM by: SCSIO Page 1 of 1

Figure S8. HRESIMS spectrum of **1**.

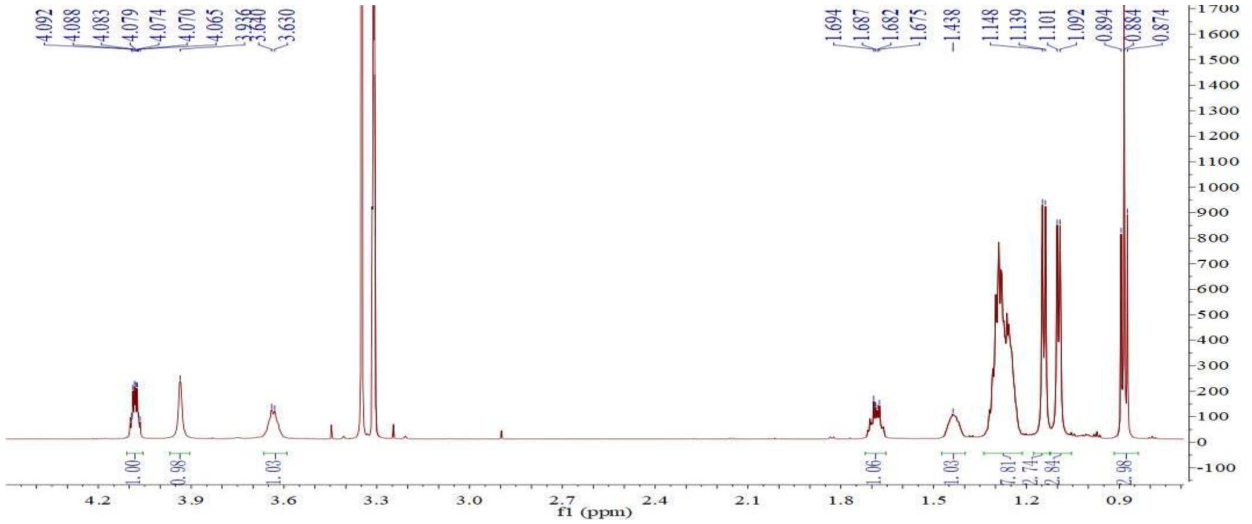

Figure S9. ^{1}H NMR spectrum of **2** in CD_3OD

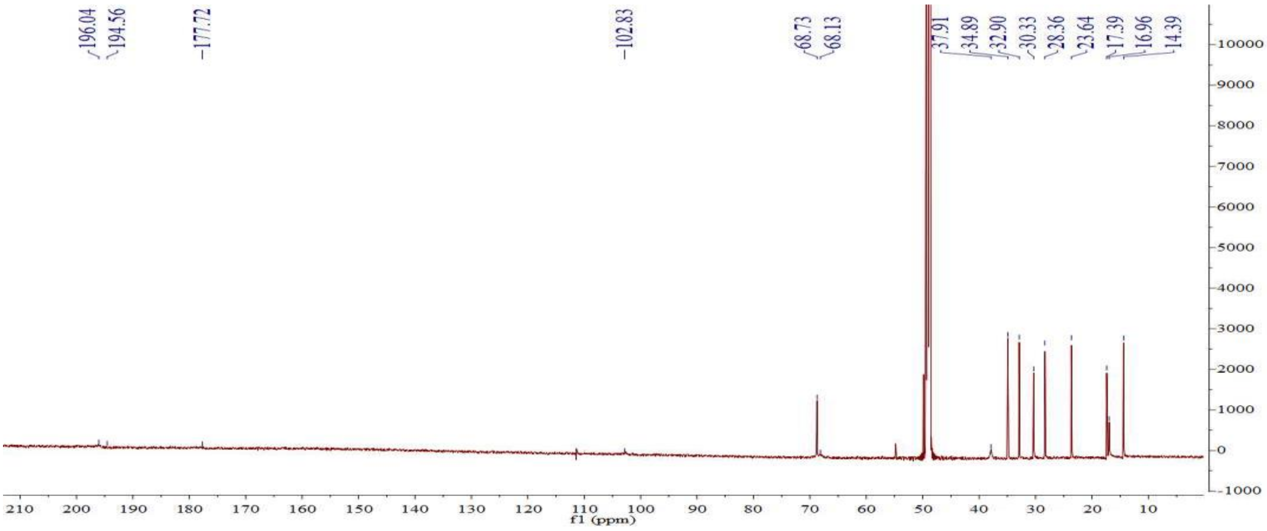

Figure S10. ^{13}C NMR spectrum of **2** in CD_3OD.

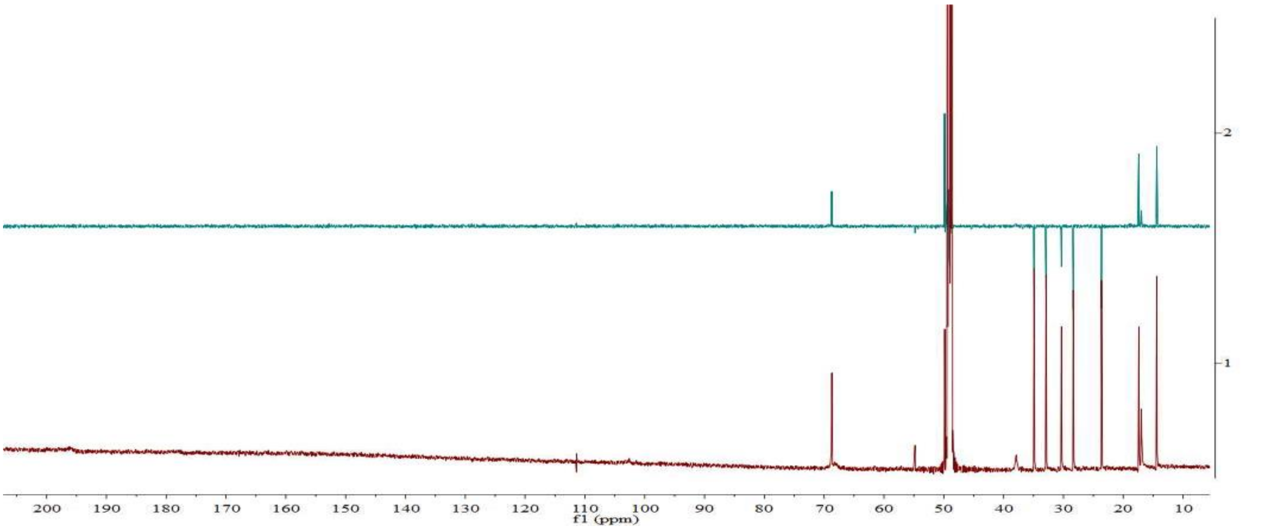

Figure S11. DEPT NMR spectrum of **2** in CD_3OD.

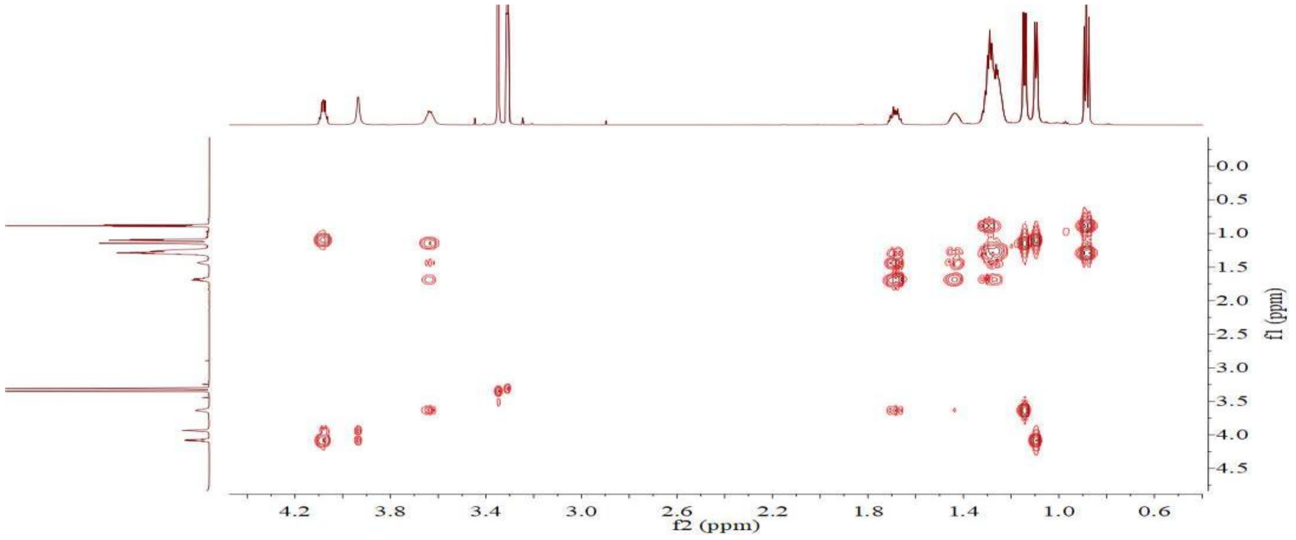

Figure S12. ^{1}H-^{1}H COSY spectrum of **2** in CD_3OD.

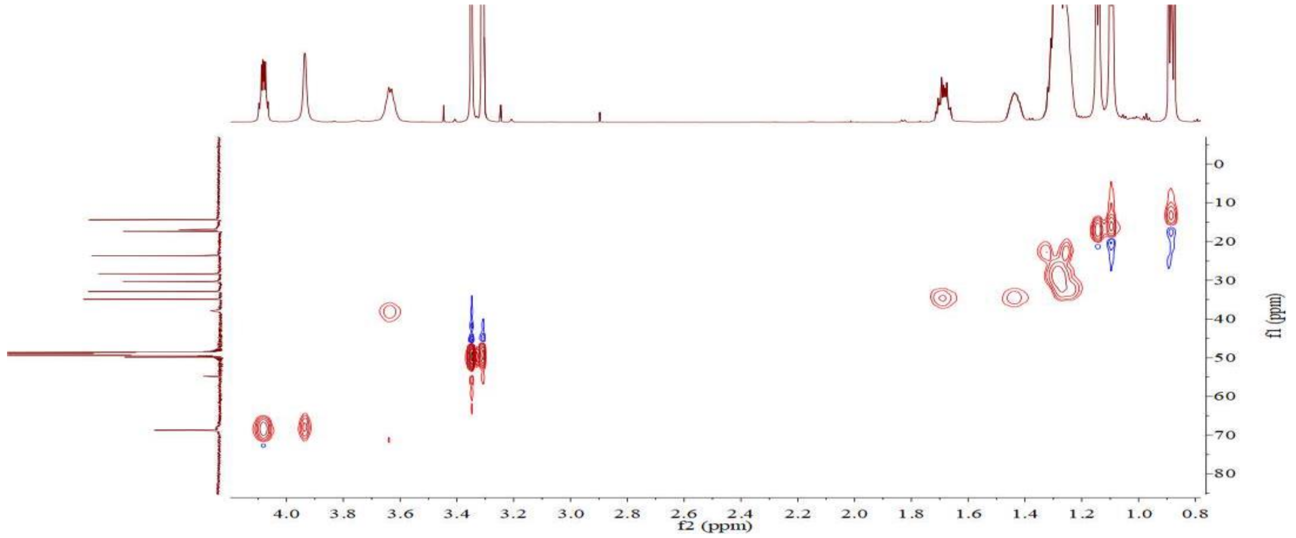

Figure S13. HSQC spectrum of **2** in CD_3OD.

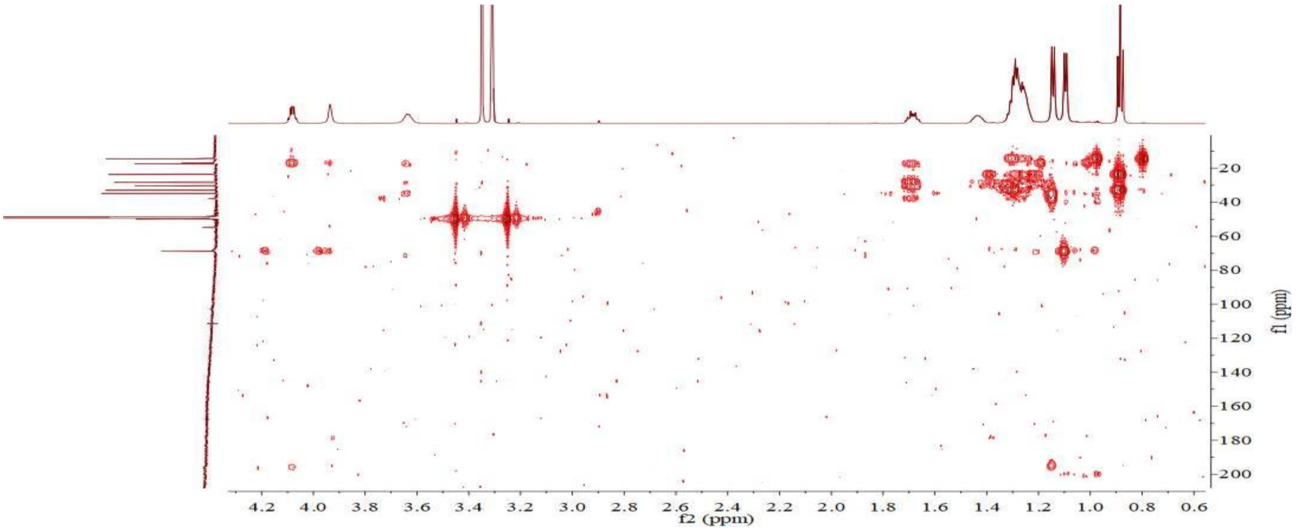

Figure S14. HMBC spectrum of **2** in CD_3OD.

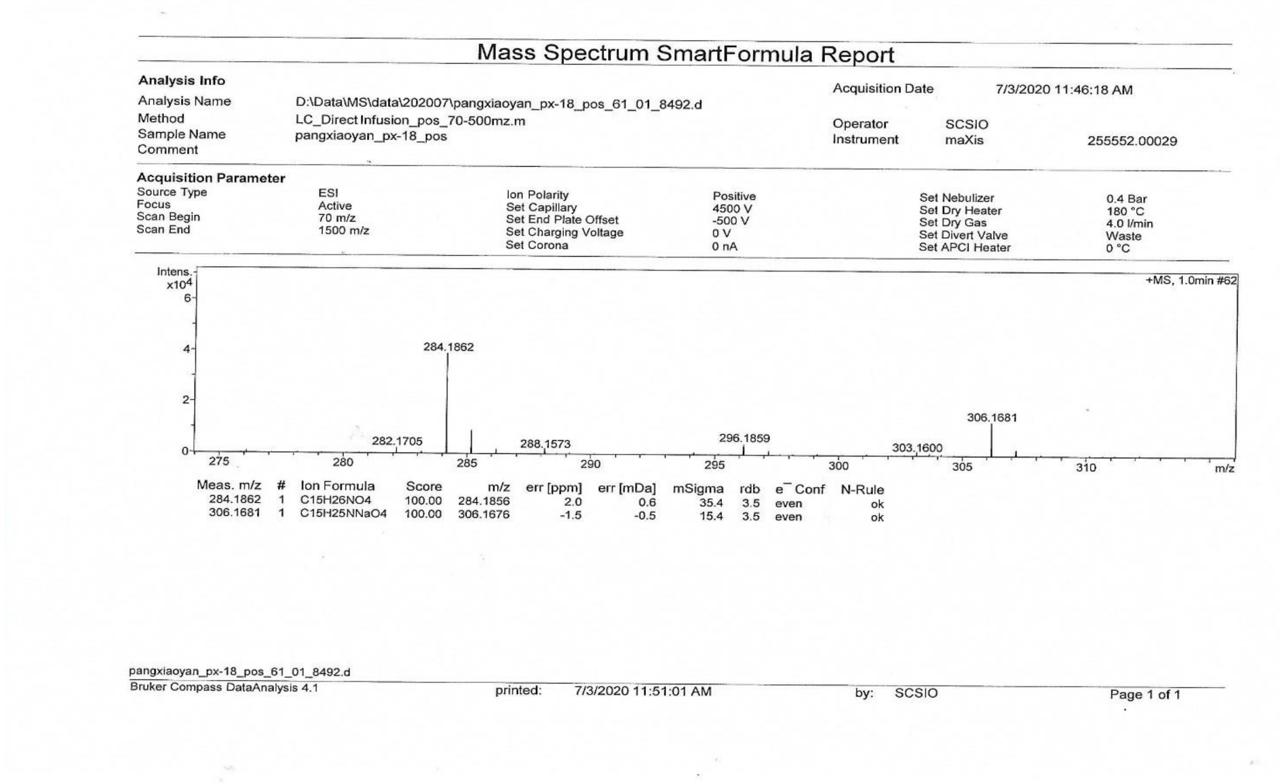

Figure S15. HRESIMS spectrum of **2**.

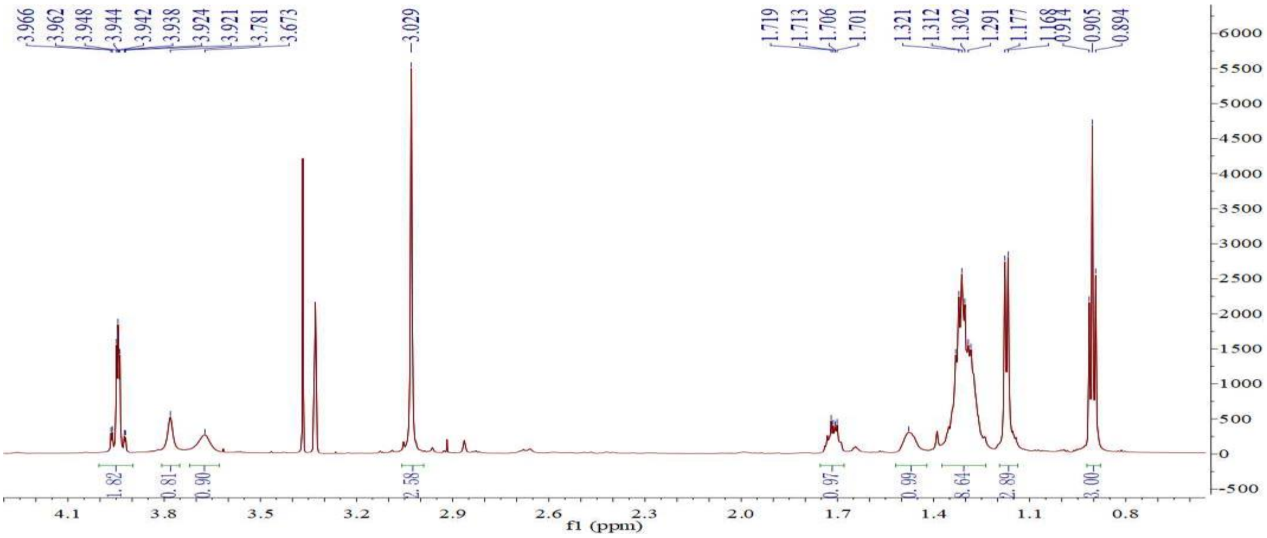

Figure S16. ^{1}H NMR spectrum of **3** in CD_3OD.

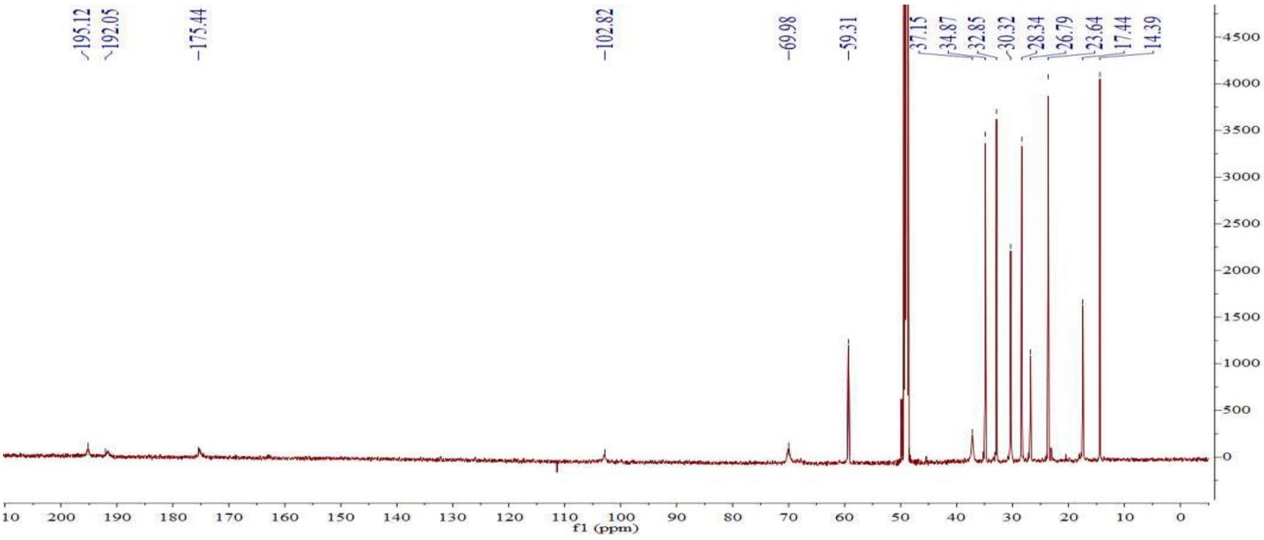

Figure S17. ^{13}C NMR spectrum of **3** in CD_3OD.

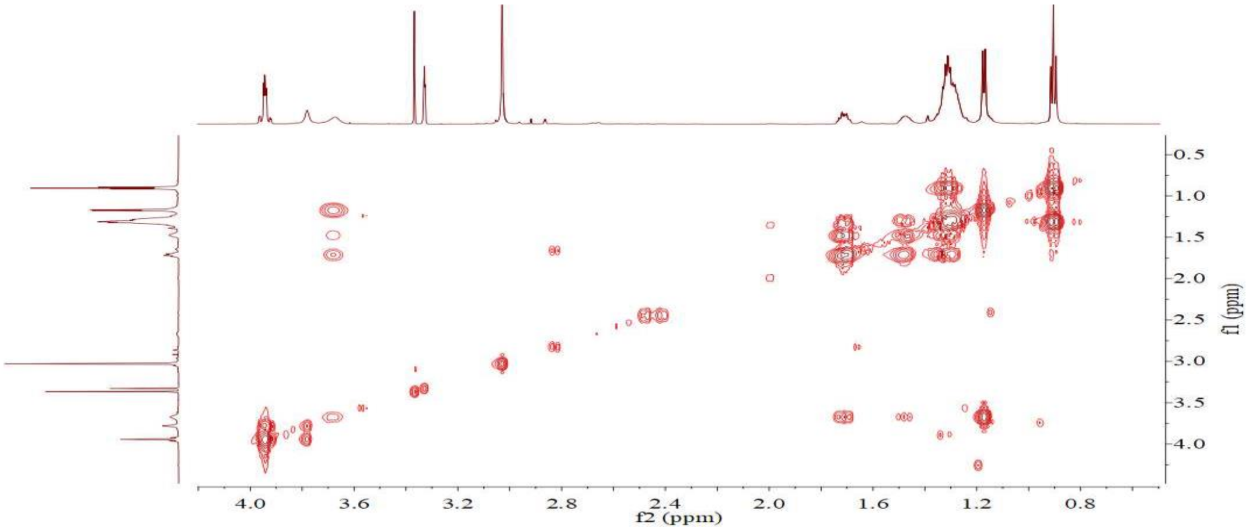

Figure S18. ^{1}H-^{1}H COSY spectrum of **3** in CD_3OD.

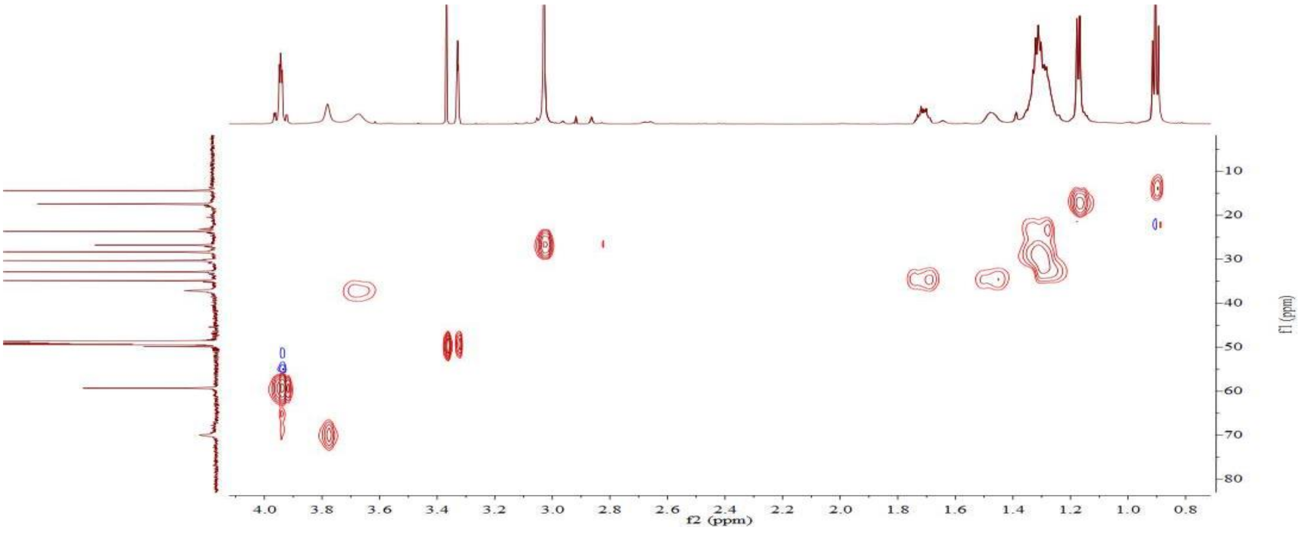

Figure S19. HSQC spectrum of **3** in CD_3OD.

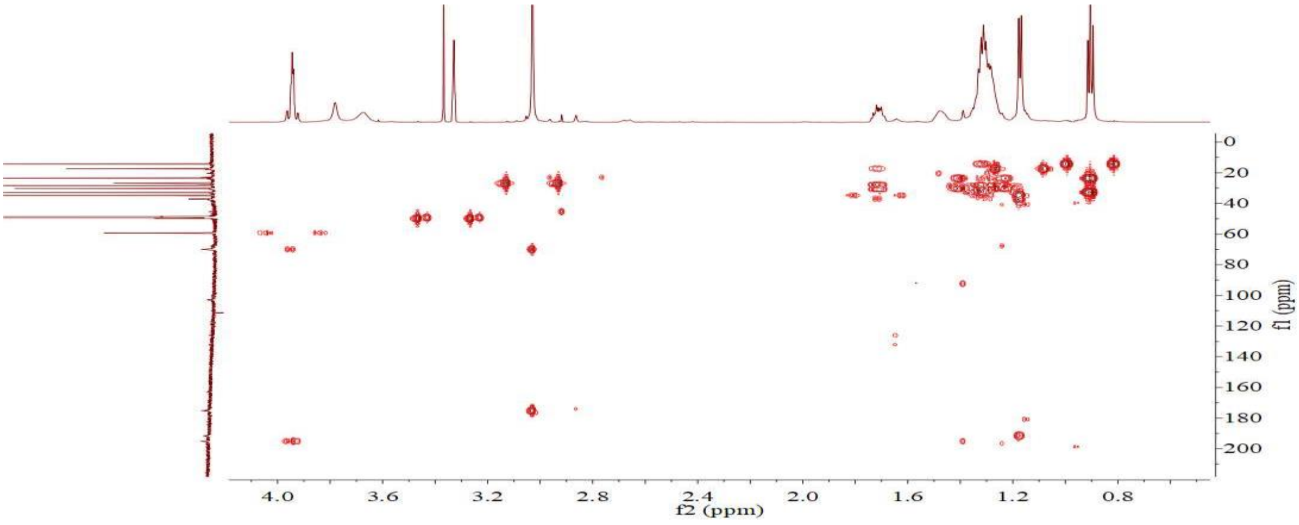

Figure S20. HMBC spectrum of **3** in CD_3OD.

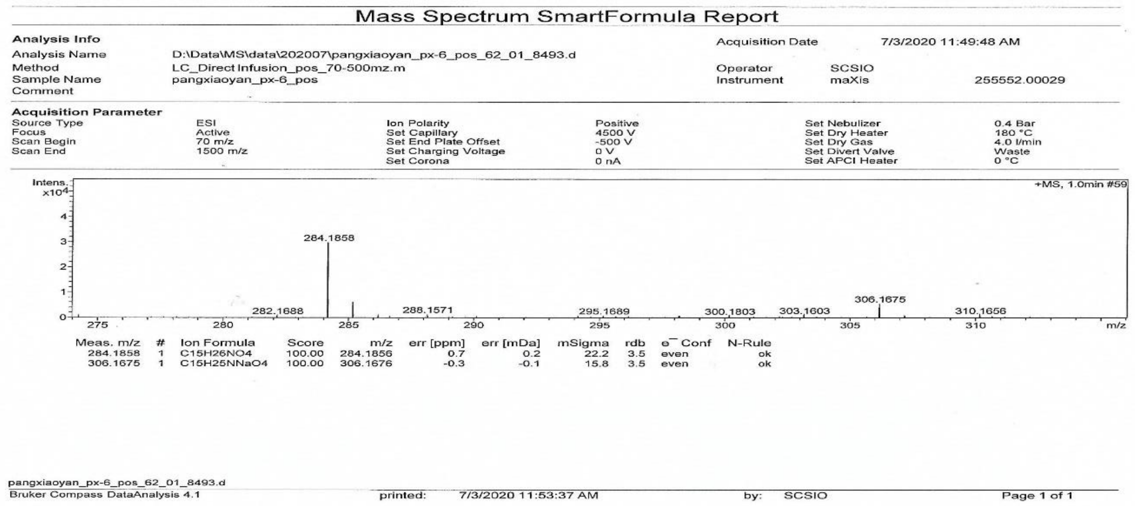

Mass Spectrum SmartFormula Report

Analysis Info
Analysis Name D:\Data\MS\data\202007\pangxiaoyan_px-6_pos_62_01_8493.d
Method LC_Direct Infusion_pos_70-500mz.m
Sample Name pangxiaoyan_px-6_pos
Comment
Acquisition Date 7/3/2020 11:49:48 AM
Operator SCSIO
Instrument maXis 255552.00029

Acquisition Parameter

Source Type	ESI	Ion Polarity	Positive	Set Nebulizer	0.4 Bar
Focus	Active	Set Capillary	4500 V	Set Dry Heater	180 °C
Scan Begin	70 m/z	Set End Plate Offset	-500 V	Set Dry Gas	4.0 l/min
Scan End	1500 m/z	Set Charging Voltage	0 V	Set Divert Valve	Waste
		Set Corona	0 nA	Set APCI Heater	0 °C

Meas. m/z	#	Ion Formula	Score	m/z	err [ppm]	err [mDa]	mSigma	rdb	e⁻ Conf	N-Rule
284.1858	1	C15H26NO4	100.00	284.1856	0.7	0.2	22.2	3.5	even	ok
306.1675	1	C15H25NNaO4	100.00	306.1676	-0.3	-0.1	15.8	3.5	even	ok

pangxiaoyan_px-6_pos_62_01_8493.d
Bruker Compass DataAnalysis 4.1 printed: 7/3/2020 11:53:37 AM by: SCSIO Page 1 of 1

Figure S21. HRESIMS spectrum of **3**.

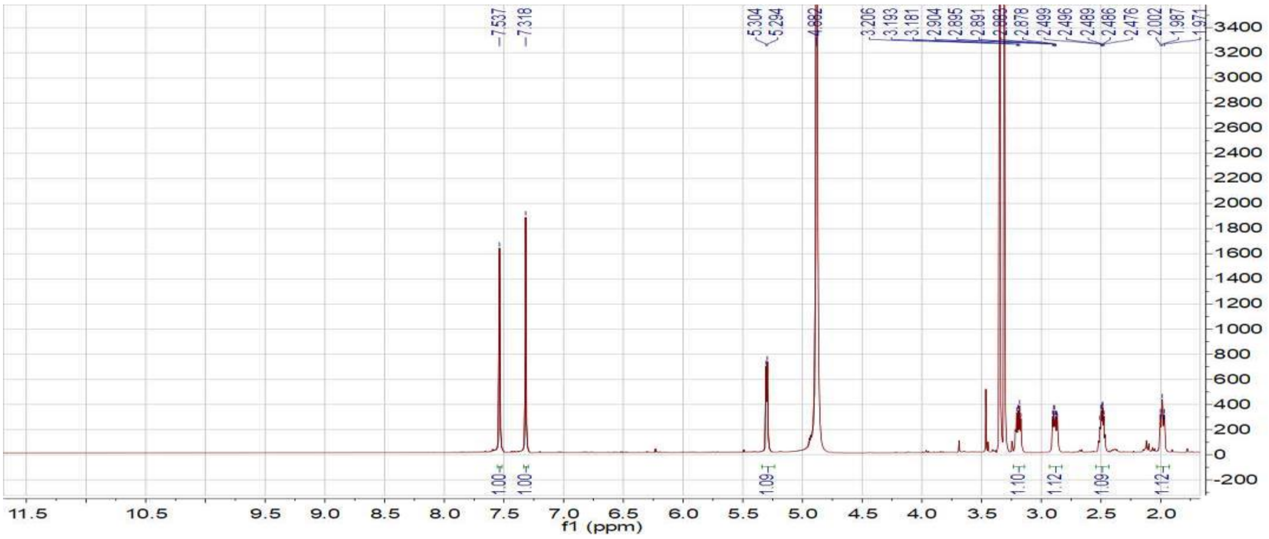

Figure S22. ^{1}H NMR spectrum of **4** in CD_3OD.

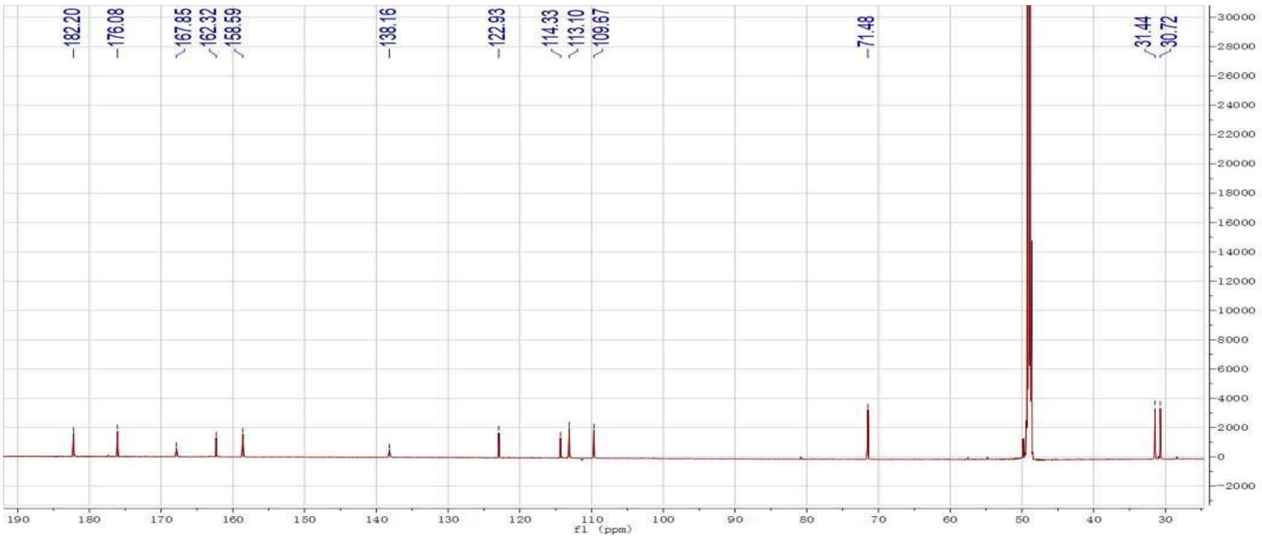

Figure S23. ^{13}C NMR spectrum of **4** in CD_3OD.

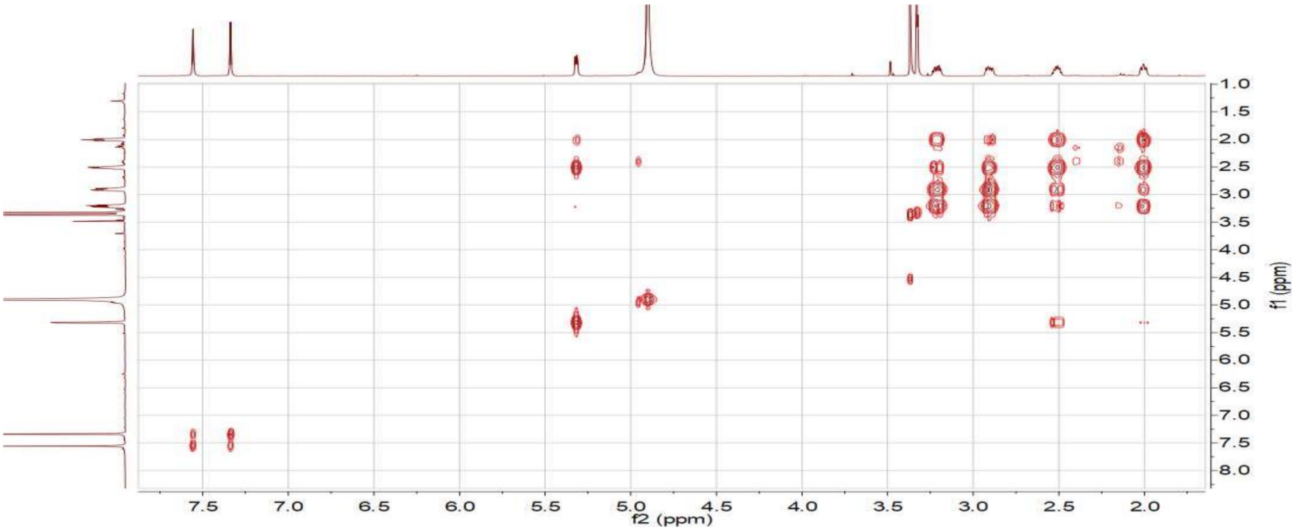

Figure S24. ^{1}H-^{1}H COSY spectrum of **4** in CD_3OD.

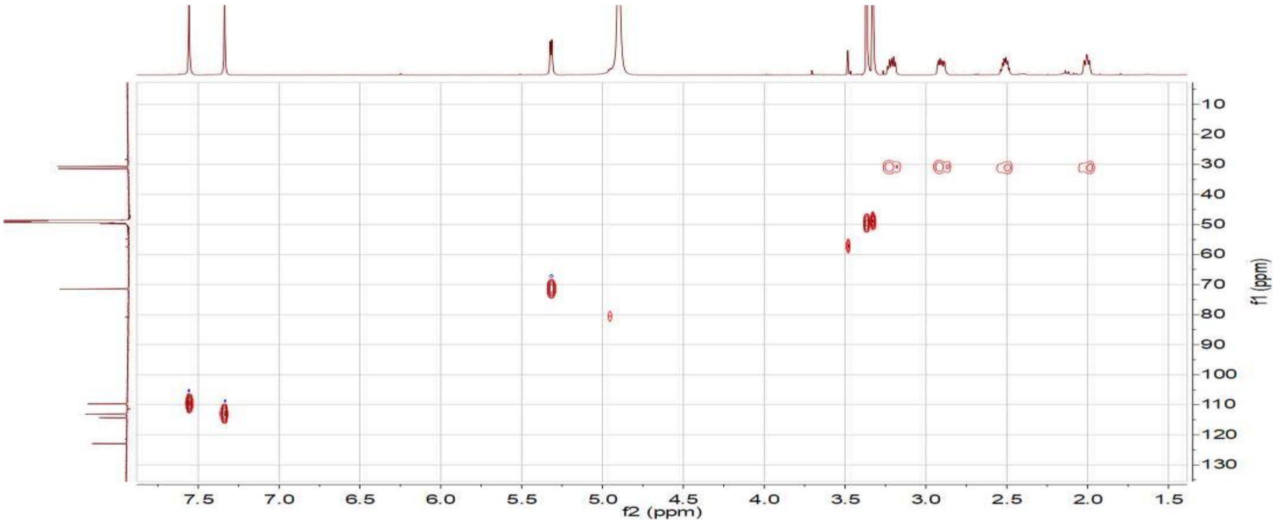

Figure S25. HSQC spectrum of **4** in CD_3OD.

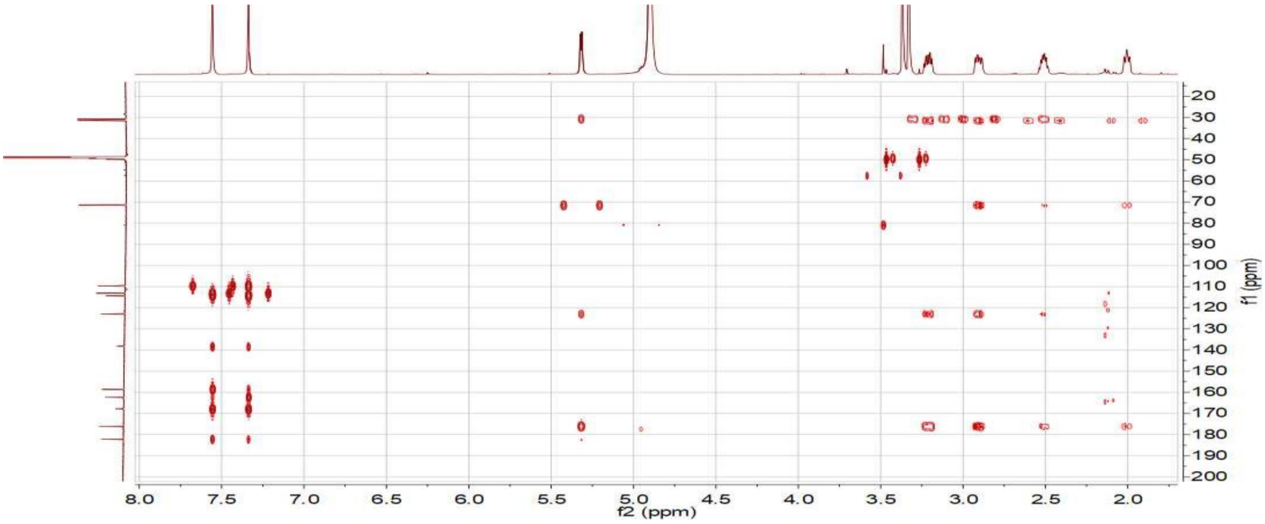

Figure S26. HMBC spectrum of **4** in CD_3OD.

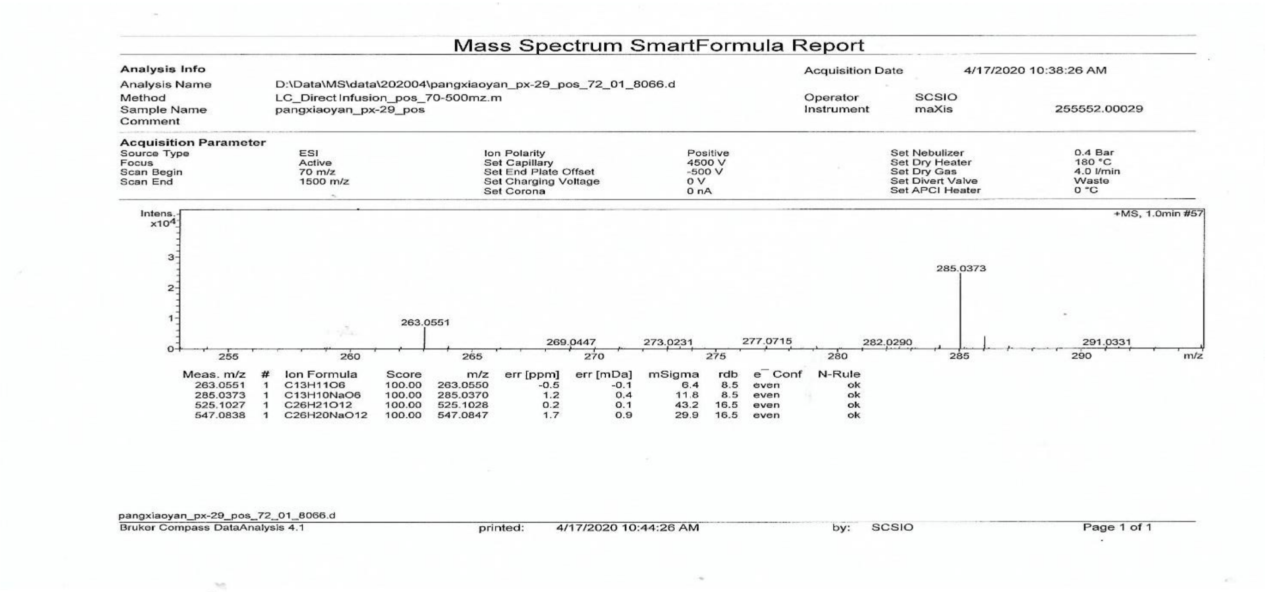

Mass Spectrum SmartFormula Report

**Analysis Info**		Acquisition Date	4/17/2020 10:38:26 AM
Analysis Name	D:\Data\MS\data\202004\pangxiaoyan_px-29_pos_72_01_8066.d		
Method	LC_Direct Infusion_pos_70-500mz.m	Operator	SCSIO
Sample Name	pangxiaoyan_px-29_pos	Instrument	maXis 255552.00029
Comment			

Acquisition Parameter

Source Type	ESI	Ion Polarity	Positive	Set Nebulizer	0.4 Bar
Focus	Active	Set Capillary	4500 V	Set Dry Heater	180 °C
Scan Begin	70 m/z	Set End Plate Offset	-500 V	Set Dry Gas	4.0 l/min
Scan End	1500 m/z	Set Charging Voltage	0 V	Set Divert Valve	Waste
		Set Corona	0 nA	Set APCI Heater	0 °C

Meas. m/z	#	Ion Formula	Score	m/z	err [ppm]	err [mDa]	mSigma	rdb	e⁻ Conf	N-Rule
263.0551	1	C13H11O6	100.00	263.0550	-0.5	-0.1	6.4	8.5	even	ok
285.0373	1	C13H10NaO6	100.00	285.0370	1.2	0.4	11.8	8.5	even	ok
525.1027	1	C26H21O12	100.00	525.1028	0.2	0.1	43.2	16.5	even	ok
547.0838	1	C26H20NaO12	100.00	547.0847	1.7	0.9	29.9	16.5	even	ok

pangxiaoyan_px-29_pos_72_01_8066.d
Bruker Compass DataAnalysis 4.1 printed: 4/17/2020 10:44:26 AM by: SCSIO Page 1 of 1

Figure S27. HRESIMS spectrum of **4**.
